# Predicting persistent fever in patients with cancer receiving antibiotics: a machine learning approach

**DOI:** 10.1038/s41746-026-03449-w

**Published:** 2026-10-31

**Authors:** Gernot Pucher, Sanjat Kanjilal, Kevin Kopp, Aman Deep, Till Rostalski, Martin Schuler, Felix Nensa, Jens Kleesiek, H. Christian Reinhardt, Christopher M. Sauer

**Affiliations:** 1 Laboratory for Clinical Research and Real-World Evidence, Department of Hematology & Stem Cell Transplantation, West German Cancer Center, University Hospital Essen, Essen, Germany; 2 Institute for Artificial Intelligence in Medicine, University Hospital Essen, Essen, Germany; 3 National Center for Tumor Diseases (NCT-West), Campus Essen, Essen, Germany; 4 Department of Medical Microbiology and Infection Prevention, Amsterdam University Medical Centers, Amsterdam, The Netherlands; 5 Brigham and Women’s Hospital, Division of Infectious Diseases, Harvard Medical School, Boston, MA USA; 6 Department of Medical Oncology, West German Cancer Center, University Hospital Essen, Essen, Germany; 7 West German Cancer Center, University Hospital Essen, Essen, Germany; 8 DKTK Partner Site Essen/Düsseldorf, University Hospital Essen, Essen, Germany; 9 Department of Physics, TU Dortmund University, Dortmund, Germany; 10 Faculty of Medicine, University of Duisburg-Essen, Essen, Germany; 11 Department of Hematology & Stem Cell Transplantation, West German Cancer Center, University Hospital Essen, Essen, Germany; 12 Center for Molecular Biotechnology, University Hospital Essen, Essen, Germany

**Keywords:** Cancer, Diseases, Health care, Medical research, Oncology

## Abstract

Persistent fever after initiating intravenous antibiotics is common in hospitalized patients with cancer and often leads to additional imaging and antimicrobial escalation. Early identification of patients unlikely to remain febrile could provide additional information during clinical reassessment. We developed and externally validated machine learning models to predict persistent fever 48–72 h after antibiotic initiation using electronic health records from two academic hospitals. Adult cancer patients with fever within 24–48 h of treatment were included. Models incorporated demographics, comorbidities, laboratory results, vital signs, and temperature patterns. The best-performing model achieved an AUROC of 0.82 (95% CI 0.79–0.84) internally and 0.78 (95% CI 0.77–0.79) externally. Temperature-related variables were the dominant predictors. Decision curve analysis demonstrated positive net benefit across much of the evaluated threshold range. Retrospective assessment suggested that approximately one quarter of antibiotic escalations and chest CT scans occurred in patients correctly predicted not to remain febrile, highlighting the potential contextual value of fever predictions. These findings indicate that short-term fever persistence can be predicted with clinically meaningful accuracy using routine data. However, clinical use should be limited to an adjunctive role rather than standalone rule-out decision-making, and prospective studies are required to evaluate safety, clinician uptake, and real-world impact.

## Introduction

Antibiotic resistance is a growing clinical and public health concern. In 2019, an estimated 4.95 million deaths worldwide were associated with antimicrobial resistance (AMR)^1^, and this number may rise to 10 million annually by 2050^2^. In Europe, the primary driver of AMR is inappropriate antibiotic use, including overuse, incorrect antibiotic selection, and suboptimal dosing^3^. Campaigns such as *Choosing Wisely* have successfully reduced unnecessary prescriptions in situations where guidelines already advocated against antibiotic prescription^4^. However, these broad strategies are less effective for critically ill patients, such as immunocompromised individuals with cancer, who require more personalized treatment approaches. Advances in electronic health records (EHRs) and analytical methods now offer opportunities for data-driven, individualized clinical decision-making in such high-risk subgroups^5^. Indeed, this is urgently needed, as large scale randomized controlled trials are not feasible to generate new insights for smaller subpopulations^6^.

Despite this progress, targeted applications for cancer patients predicting short-term clinical outcomes such as persistent fever remain limited. Cancer patients represent a distinct high-risk population due to multiple factors, including disease-related immunodeficiency, therapy-induced leukopenia and immune modulation, and overall reduced health. Although clinical risk scores such as MASCC, CISNE, and Talcott’s rules assist in stratifying the risk of serious complications in febrile neutropenia, these tools do not predict the short-term trajectory of fever after antibiotic initiation^7^. Because fever in this group has diverse etiologies^8^, clinical guidelines typically recommend empiric broad-spectrum antibiotics and reassessment at 48–72 h, including consideration of CT imaging to evaluate viral, fungal, or other non-bacterial causes of persistent fever. While this management strategy prioritizes safety, it may inadvertently contribute to additional antibiotic exposure and diagnostic procedures^9^. A targeted predictive model estimating the likelihood of fever persistence between 48 and 72 h after initiating antibiotic therapy could support a more individualized approach. While it would not determine the underlying cause of fever or provide direct treatment recommendations, its predictions could serve as an additional signal during reassessment, complementing clinical judgement. The potential of machine learning algorithms (ML) for augmenting the role of physicians and improving healthcare quality is widely acknowledged and backed by successful clinical implementations^10^. EHRs provide the extensive, structured datasets needed to train these models^11^. Numerous studies have applied AI and ML models to EHRs to predict outcomes like sepsis, using a range of models including ensemble methods, deep learning, and more recently, large language models (LLMs)^12^.

This study aims to (a) develop and validate ML models to predict fever persistence in cancer patients 48 h after the initiation of antibiotic therapy, (b) explore the relationship between model predictions and treatment escalation and diagnostic imaging and (c) assess the generalizability and robustness of these models through external validation in a different hospital setting.

## Results

### Cohort building

**Fig. 1:**
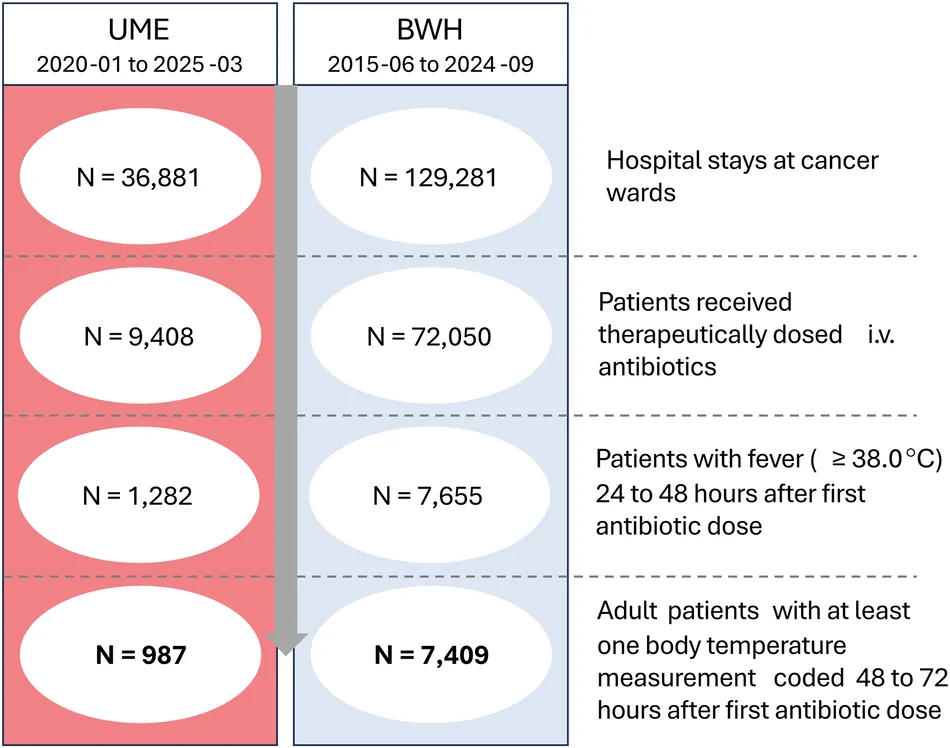
Flow diagram illustrating the cohort extraction steps for the UME and BWH datasets. UME = University Medicine Essen, Germany; BWH = Brigham and Women’s Hospital, USA.

Among 36,881 hospital admissions of cancer patients at University Medicine Essen (UME) between January 2020 and March 2025, antibiotic therapy was administered in 9408 cases. At Brigham and Women’s Hospital (BWH), 129,281 admissions of cancer patients were identified between June 2015 and September 2024, of which 72,050 included antibiotic treatment. Fever during 24–48 h of antibiotic treatment was observed in 1282 cases (13.6%) in the UME cohort and 7655 cases (10.6%) in the BWH cohort. Restricting to adult patients with at least one documented temperature measurement between 48 and 72 h after initiation of antibiotic therapy, which were required for outcome determination, resulted in final cohorts of 987 hospital stays among 794 unique patients at UME and 7409 hospital stays among 6256 unique patients at BWH (Fig. 1). The distribution of hospital stays per patient is shown in Supplementary Table 1. Among the 244 UME hospital stays excluded because of unavailable temperature measurements during the outcome window, 27.9% (68/244) showed a temporary monitoring gap and 26.2% (64/244) had no subsequent temperature documentation. Other reasons for missing body temperature documentation included death, discharge, ICU transfer, and ward transfer (Supplementary Table 2). Detailed distributions of maximum body temperature are provided in Supplementary Table 3.

The training and internal validation cohort from UME and the external validation cohort from BWH showed variations in their characteristics (Table 1). The proportion of hospital stays with persistent fever 48–72 h after initiation of antibiotic therapy differed numerically between cohorts, with 54.0% (533/987) in UME and 46.5% (3442/7409) in BWH, reflecting expected variation in baseline risk across sites. Furthermore, the UME cohort was dominated by leukemia (31.4%, 310/987) and other hematologic cancers (51.0%, 503/987), whereas the BWH cohort included a larger proportion of patients with solid tumors (56.8%, 4209/7409).

**Table 1:** Baseline characteristics of cancer patient hospitalizations at the University Medicine Essen and Brigham and Women’s Hospital, grouped by persistent fever status 48 to 72 h after initiation of antibiotic therapy

	University Medicine Essen		Brigham and Women’s Hospital	
	Fever	No Fever	Fever	No Fever
Hospitalizations				
Hospital stays, *n* (%)	533 (54.0)	454 (46.0)	3442 (46.5)	3967 (53.5)
Length of stays until first admin. of antibiotics, d, mean (SD)	6.5 (5.9)	6.1 (6.4)	3.0 (2.8)	2.8 (2.5)
Demographics				
Age of patients, mean (SD)	53.5 (16.0)	55.3 (16.1)	59.0 (15.1)	61.9 (14.9)
Female sex, *n* (%)	193 (36.2)	186 (41.0)	1394 (40.5)	1762 (44.4)
Conditions				
Elixhauser score of patients, mean (SD)	7.7 (3.0)	7.6 (3.0)	7.2 (3.1)	7.1 (3.2)
Main cancer diagnosis, *n* (%)				
Leukemia	177 (33.2)	133 (29.3)	1049 (30.5)	715 (18.0)
Other hematological malignancies	273 (51.2)	230 (50.7)	748 (21.7)	688 (17.3)
Solid tumors	83 (15.6)	91 (20.0)	1645 (47.8)	2564 (64.6)
Lab. values 24–48 h after first AB				
Creatinine, mg/dl, mean (SD)	0.9 (0.5)	1.0 (0.6)	1.1 (0.9)	1.1 (1.0)
CRP, mg/dl, mean (SD)	16.4 (9.6)	14.7 (9.5)	10.1 (4.9)	9.9 (4.2)
Bilirubin, mg/dl, mean (SD)	1.0 (0.8)	1.1 (1.3)	1.0 (1.5)	1.1 (1.7)
Leukocytes, nl, mean (SD)	8.5 (25.5)	6.2 (13.8)	10.8 (20.9)	10.8 (15.1)
Hemoglobin, g/dl, mean (SD)	8.9 (1.6)	9.0 (1.6)	9.4 (1.7)	9.7 (1.8)
Vital signs 24–48 h after first AB				
Mean arterial pressure, mmHg, mean (SD)	88.2 (10.4)	87.4 (10.8)	94.6 (14.7)	94.1 (12.8)
Heart rate, bpm, mean (SD)	93.5 (10.0)	92.4 (8.9)	108.5 (18.3)	104.4 (18.3)
Pulse oximetry SpO2, %, mean (SD)	96.5 (1.8)	96.6 (1.4)	98.3 (1.7)	98.2 (1.7)
Body temperature, °C				
Highest body temp. 24–48 h after first AB, mean (SD)	38.8 (0.6)	38.4 (0.4)	38.8 (0.6)	38.5 (0.5)
Highest body temp. 0–24 h after first AB, mean (SD)	38.7 (0.7)	38.4 (0.8)	38.6 (0.8)	38.3 (0.8)
Recordings of body temp. 24–48 h after first AB, mean (SD)	5.9 (2.3)	5.2 (1.6)	11.6 (9.5)	10.2 (9.2)
Recordings of body temp. 0–24 h after first AB, mean (SD)	6.4 (2.3)	5.8 (2.2)	12.7 (10.4)	11.9 (10.4)

Treatment and monitoring patterns varied between the two centers as well. Patients in the UME cohort experienced longer intervals between hospital admission and first antibiotic administration (approximately 6 days vs. 3 days in the BWH cohort). Temperature monitoring was also considerably more frequent in the BWH cohort, with an average of 12.2 (SD 10.4) and 10.8 (SD 9.4) measurements taken within 0–24 and 24–48 h after antibiotic initiation, respectively, compared with 6.1 (SD 2.3) and 5.6 (SD 2.0) measurements in the UME cohort.

Physiologic and laboratory parameters showed comparable value ranges across cohorts, with the most notable differences observed in the inflammatory markers C-reactive protein (UME: 15.6 mg/dl, SD 9.6; BWH: 10.0 mg/dl, SD 4.5) and leukocyte counts (UME: 7.4 nl, SD 21.0; BWH: 10.8 nl, SD 18.0).

### Model performance

Across both internal and external evaluations, all ML models demonstrated improved discrimination, calibration characteristics, and threshold-dependent utility compared with the naïve model. While performance varied modestly between models, overall patterns were consistent across datasets, and the advanced models maintained stable discrimination when applied to the external cohort.

**Fig. 2:**
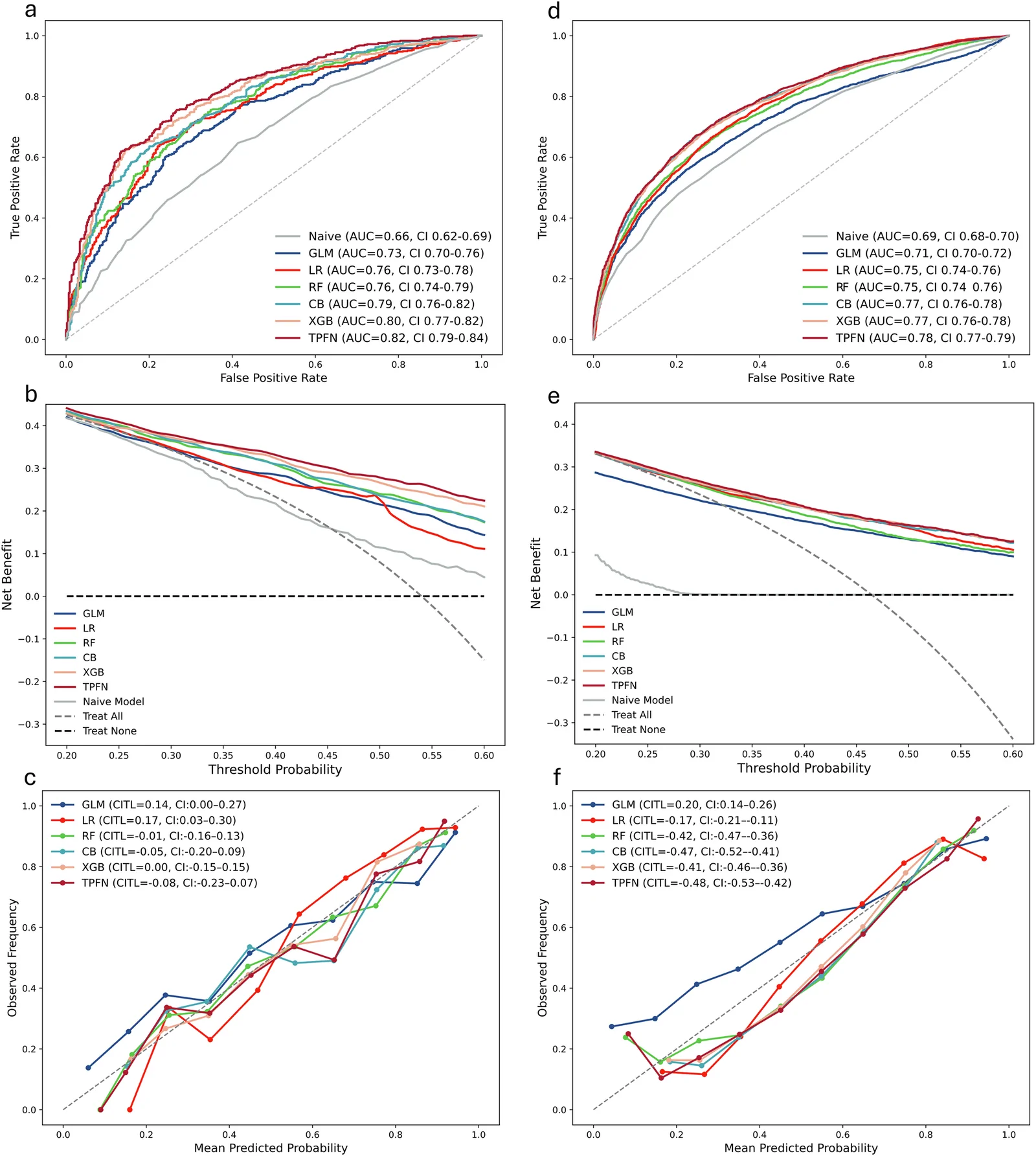
Discrimination, clinical utility, and calibration of prediction models in the internal (UME) and external (BWH) validation cohorts. **a**–**c** show model performance in the internal UME dataset, and **d**–**f** show performance in the external BWH dataset. **a**, **d** Receiver operating characteristic (ROC) curves with bootstrapped AUROC estimates (95% CI) for all models, comparing a naïve baseline with generalized linear and machine learning approaches. **b**, **e** Decision curve analysis (DCA) demonstrating net benefit across threshold probabilities (0.20–0.60) relative to Treat All and Treat None strategies. **c**, **f** Calibration plots depicting agreement between predicted and observed risk, with calibration-in-the-large (CITL) and corresponding 95% confidence intervals shown for each model. UME = University Medicine Essen; BWH = Brigham and Women’s Hospital; Naïve = last value forward baseline predictor; GLM = Generalized linear model; LR = Regularized logistic regression; RF = Random Forest; XGB = XGBoost; CB = CatBoost; TPFN = TabPFN.

In the internal validation using nested 5-fold cross-validation on the UME dataset (Fig. 2a), all models outperformed the naïve baseline (AUROC 0.66, 95% CI 0.62–0.69). Regularized logistic regression, Random Forest, XGBoost and CatBoost achieved AUROCs between 0.76 and 0.80. TabPFN showed the highest point estimate at 0.82 (95% CI 0.79–0.84), although confidence intervals overlapped with those of other ML models.

In the external BWH cohort (Fig. 2d), AUROC values were generally consistent with internal validation. The generalized linear model reached 0.71 (95% CI 0.70–0.72), and the ML models achieved AUROCs between 0.75 and 0.77. TabPFN again yielded the highest point estimate at 0.78 (95% CI 0.77–0.79).

Across both the UME (Fig. 2b) and BWH (Fig. 2e) cohorts, model-based strategies showed positive net benefit across much of the evaluated threshold range. Incremental net benefit over the treat-all strategy was small at lower threshold probabilities, however. Differences in net-benefit profiles among ML models were modest, with no single model showing a clear advantage across threshold ranges.

Most models demonstrated good correspondence between predicted and observed risks across the probability range in the UME cohort (Fig. 2c), with calibration-in-the-large (CITL) values close to zero. In the external cohort (Fig. 2f), all models except for the generalized linear model showed a systematic shift toward overprediction, directionally consistent with the lower outcome prevalence in the BWH dataset relative to the training cohort (46% vs. 54%). Although the overall shapes of the calibration curves were similar across models, the observed calibration shift suggests that model recalibration may be necessary before deployment in settings with different outcome prevalence.

Threshold-based metrics further supported these findings. In both the internal (Table 2a) and the external (Table 2b) cohort, ML models consistently outperformed the baseline models, which showed moderately high specificity but low sensitivity in both settings. Although confidence intervals overlapped with those of XGBoost and CatBoost, TabPFN’s point estimates were consistently among the highest across specificity (0.78, 95% CI 0.74–0.81), sensitivity (0.70, 95% CI 0.65–0.73), and F1 score (0.74, 95% CI 0.70–0.77) in the UME cohort. Its performance remained robust in the external cohort, with specificity of 0.76 (95% CI 0.74–0.77), sensitivity of 0.69 (95% CI 0.67–0.70) and an F1 score of 0.70 (95% CI 0.68–0.71). XGBoost and CatBoost demonstrated comparable performance with stable trade-off across cohorts, e.g., with specificities of 0.75 (0.71–0.79) and 0.76 (0.72–0.80) on internal validation, and of 0.74 (95% CI 0.73–0.76) on external validation. Overall, threshold-based performance patterns remained consistent between datasets.

**Table 2:** Selected threshold-based performance metrics for all models in the (a) internal validation cohort (UME) and the (b) external validation cohort (BWH)

a					
Model	F1_95% CI_	Spec. _95% CI_	Sens. _95% CI_	PPV_95% CI_	NPV_95% CI_
Naïve	0.49 (0.44–0.53)	0.81 (0.78–0.85)	0.37 (0.33–0.41)	0.70 (0.65–0.75)	0.52 (0.49–0.56)
GLM	0.68 (0.65–0.71)	0.70 (0.66–0.74)	0.63 (0.59–0.67)	0.72 (0.68–0.76)	0.63 (0.59–0.67)
LR	0.72 (0.69–0.75)	0.74 (0.70–0.78)	0.69 (0.65–0.72)	0.76 (0.72–0.80)	0.67 (0.63–0.71)
RF	0.69 (0.66–0.72)	0.71 (0.67–0.75)	0.66 (0.62–0.70)	0.73 (0.69–0.77)	0.64 (0.60–0.68)
XGB	0.73 (0.70–0.76)	0.75 (0.71–0.79)	0.70 (0.66–0.73)	0.76 (0.73–0.80)	0.68 (0.64–0.72)
CB	0.73 (0.70–0.76)	0.76 (0.72–0.80)	0.70 (0.66–0.73)	0.77 (0.73–0.81)	0.68 (0.64–0.72)
TPFN	0.74 (0.70–0.77)	0.78 (0.74–0.81)	0.70 (0.65–0.73)	0.79 (0.74–0.82)	0.68 (0.64–0.72)

b					
Model	F1_95% CI_	Spec. _95% CI_	Sens. _95% CI_	PPV_95% CI_	NPV_95% CI_
Naïve	0.45 (0.43–0.47)	0.88 (0.87–0.89)	0.33 (0.31–0.34)	0.71 (0.69–0.73)	0.60 (0.59–0.62)
GLM	0.62 (0.60–0.63)	0.78 (0.77–0.79)	0.56 (0.54–0.57)	0.69 (0.67–0.70)	0.67 (0.66–0.68)
LR	0.67 (0.66–0.68)	0.72 (0.70–0.73)	0.66 (0.65–0.68)	0.67 (0.66–0.69)	0.71 (0.70–0.73)
RF	0.66 (0.65–0.68)	0.72 (0.70–0.73)	0.66 (0.64–0.67)	0.67 (0.65–0.69)	0.71 (0.69–0.72)
XGB	0.69 (0.67–0.70)	0.74 (0.73–0.76)	0.68 (0.67–0.70)	0.70 (0.68–0.71)	0.73 (0.71–0.74)
CB	0.69 (0.68–0.70)	0.74 (0.73–0.76)	0.69 (0.67–0.70)	0.70 (0.68–0.71)	0.73 (0.72–0.74)
TPFN	0.70 (0.68–0.71)	0.76 (0.74–0.77)	0.69 (0.67–0.70)	0.70 (0.69–0.72)	0.73 (0.71–0.74)

Sensitivity (Sens.), specificity (Spec.), positive predictive value (PPV), negative predictive value (NPV), and F1 score are reported with bootstrapped 95% confidence intervals. Metrics were calculated at the Youden-optimal threshold determined within the internal dataset.

*UME* University Medicine Essen, *BWH* Brigham and Women’s Hospital, *Naïve* last value forward baseline predictor, *GLM* Generalized linear model, *LR* Regularized logistic regression, *RF* Random Forest, *XGB* XGBoost, *CB* CatBoost, *TPFN* TabPFN.

At operating points targeting sensitivities of ≥90%, ≥95%, and ≥98% for the UME cohort, corresponding NPVs of the XGBoost model were 0.77 (95% CI 0.71–0.82), 0.80 (95% CI 0.74–0.86), and 0.82 (95% CI 0.73–0.89), while false-negative rates were 10.0% (95% CI 7.6–12.5%), 6.0% (95% CI 4.1–8.1%), and 3.0% (95% CI 1.7–4.5%), respectively. This came at the cost of decreasing specificity from 0.38 (95% CI 0.34–0.43) to 0.16 (95% CI 0.12–0.19) and fewer patients being classified as low risk, from 22.9% (95% CI 20.4–25.6%) to 8.8% (95% CI 7.1–10.7%).

Incorporating the continuous forecasted temperature improved discriminative performance across models in the UME dataset (e.g., TabPFN AUC 0.82 [95% CI 0.79–0.84] vs. 0.79 [0.76–0.81] without this feature; paired DeLong *p* = 0.001). In contrast, using dichotomized forecasted values from Chronos-2 as a binary fever indicator resulted in lower performance (AUC 0.74 [0.71–0.77]) (Supplementary Table 4). The forecasting of Chronos-2 achieved an MAE of 0.78 (95% CI 0.76–0.81) at UME and 0.72 (95% CI 0.71–0.73) at BWH and showed lower MAE than both the seasonal-naïve and AutoARIMA benchmarks in both cohorts.

Additional performance metrics, including regression metrics of the Chronos-generated body temperature forecasts, are reported in Supplementary Tables 5–7.

### Feature importance

**Fig. 3:**
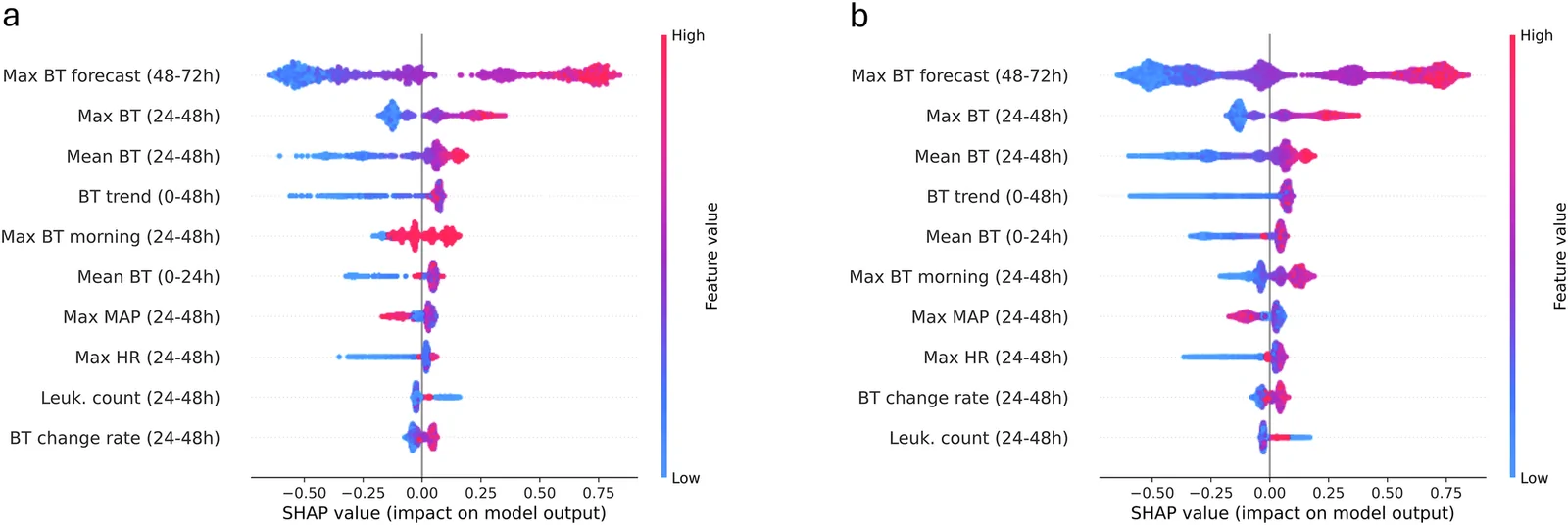
SHAP values for the top 10 predictors based on the XGBoost model. Each dot represents a hospital stay in the internal (**a**) and external (**b**) validation cohorts, with color indicating the feature value (blue = low, red = high). Features are ordered by mean absolute SHAP value. The time indications are relative to the initiation of antibiotic therapy. BT = Body temperature; Leuk. count = Leukocyte counts; MAP = Mean arterial pressure; HR = Heart rate.

To evaluate model explainability, SHAP values were computed for both the internal and external validation cohorts. Feature rankings showed moderate-to-very-high agreement between UME and BWH for the same model (Spearman 0.66–0.98; Jaccard 0.54–1.00). In contrast, agreement across different model families was limited to moderate, with mean Spearman correlations of 0.40 and 0.42 and mean Jaccard indices of 0.36 and 0.35 in UME and BWH, respectively (Supplementary Table 8). Body temperature–derived variables consistently ranked among the most influential predictors (Fig. 3). The forecast-derived maximum body temperature was the highest-ranked feature for most models and ranked first for TabPFN in both the internal and external cohorts. Other highly ranked temperature-derived features included the mean and maximum measured temperature during 24–48 h, the last temperature measured before the 48-h prediction cutoff, the proportion of temperature measurements ≥38.0 °C during 24–48 h, and the overall temperature trend during 0–48 h after antibiotic initiation. Physiological variables, including heart rate and mean arterial pressure, and laboratory parameters were also represented among the higher-ranked predictors in some models, although generally below temperature-derived features. We additionally assessed permutation feature importance to quantify the decrease in model performance when individual features are perturbed. Consistent with the SHAP results, the forecast-derived temperature feature showed the largest impact on model performance across models and datasets, e.g., with a mean decrease in AUC of 0.08 (95% CI 0.06–0.10) in the internal dataset and 0.08 (95% CI 0.07–0.09) in the external dataset for the XGBoost model. Other temperature-derived features had considerably smaller effects (e.g., XGBoost, maximum body temperature 24–48 h after initiation of antibiotic therapy, AUC 0.02 (95% CI 0.01–0.03) in the internal dataset and 0.04 (95% CI 0.03–0.04) in the external dataset), while non-temperature features contributed only marginally. Full permutation importance results are provided in Supplementary Table 9.

Temperature-focused analyses showed AUROCs of 0.70 (95% CI 0.66–0.73) and 0.72 (95% CI 0.70–0.73) for the temperature-summary logistic model, 0.73 (95% CI 0.70–0.76) and 0.74 (95% CI 0.73–0.75) for forecast-only XGBoost, and 0.57 (95% CI 0.54–0.61) and 0.56 (95% CI 0.55–0.58) for non-temperature XGBoost in UME and BWH, respectively (Supplementary Table 10).

### Error analysis

**Fig. 4:**
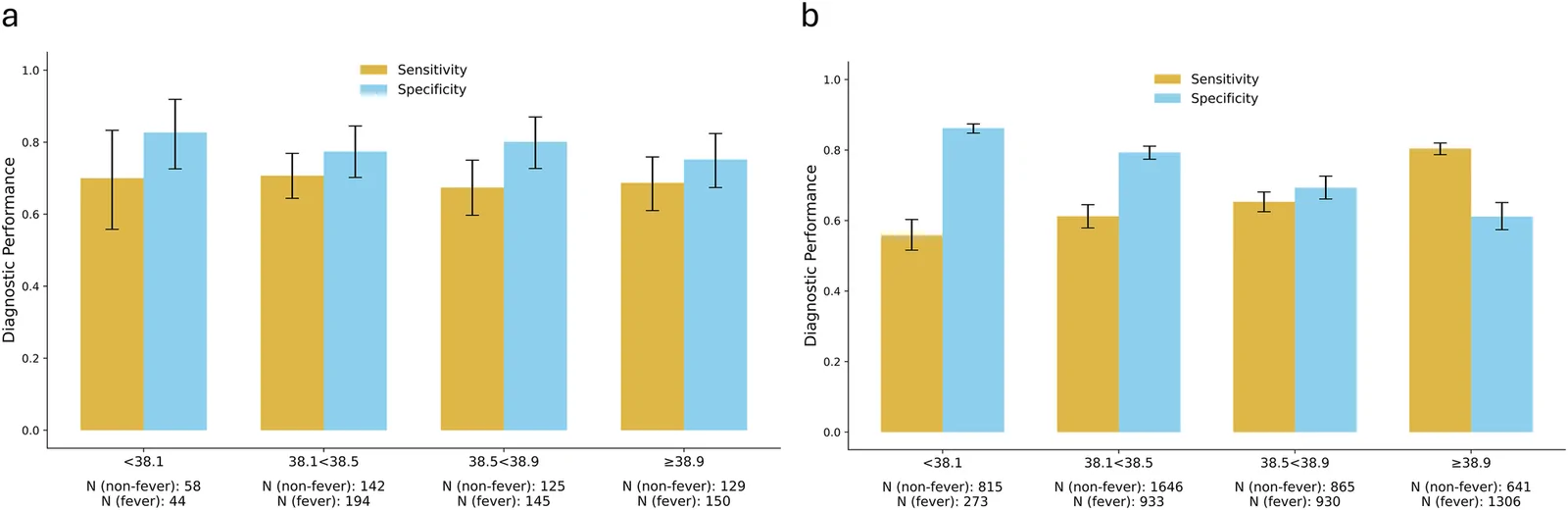
Stratified diagnostic performance of the TabPFN model by highest observed body temperature at 24–48 h of antibiotic therapy. Bars show sensitivity (orange) and specificity (blue) with 95% confidence intervals across four temperature strata in the internal (**a**) and external (**b**) cohorts. Sample sizes for fever and non-fever cases are provided below each bar group.

To ensure robust model performance across clinically relevant subgroups, we conducted stratified analyses across patient characteristics (e.g., sex, age, cancer type), which demonstrated no meaningful variation in predictive accuracy (Supplementary Tables 11–12). In contrast, stratification by the degree of fever present 24 to 48 h after initiation of antibiotic therapy revealed substantial heterogeneity in threshold-based classification performance in the external validation cohort, whereas no comparable pattern was observed in internal cross-validation (Fig. 4).

In the internal dataset, diagnostic performance remained stable across all temperature strata. Sensitivity ranged from 0.67 (95% CI 0.60–0.75) to 0.71 (95% CI 0.64–0.77), and specificity ranged from 0.75 (95% CI 0.67–0.82) to 0.83 (95% CI 0.73–0.92), with no systematic association between temperature level and model performance. In contrast, the external cohort exhibited a temperature-dependent gradient. Sensitivity rose from 0.56 (95% CI 0.52–0.60) among patients with highest observed temperatures <38.1 °C to 0.80 (95% CI 0.79–0.82) for temperatures ≥38.9 °C, whereas specificity declined from 0.86 (95% CI 0.85–0.87) to 0.61 (95% CI 0.57–0.65) across the same intervals.

### Sensitivity analysis

Model performance was robust to changes in the operational definition of fever. When the fever threshold was increased from ≥ 38.0 °C to ≥38.3 °C and the models were retrained accordingly, predictive performance remained largely unchanged across all algorithms (Supplementary Table 13). For example, the TabPFN model achieved an AUC of 0.80 (95% CI 0.77–0.83), a sensitivity of 0.69 (95% CI 0.64–0.73), and a specificity of 0.76 (95% CI 0.72–0.79) during nested cross-validation on the internal dataset—values closely mirroring those obtained under the original fever definition. External validation similarly confirmed performance stability, with an AUC of 0.79 (95% CI 0.78–0.80), a sensitivity of 0.65 (95% CI 0.63–0.67), and a specificity of 0.80 (95% CI 0.79–0.81).

Given the substantially higher frequency of temperature measurements in the BWH cohort, persistent-fever prevalence was recalculated after standardizing BWH measurement density to the empirical UME distribution. Prevalence decreased from 46.5% to a median of 43.7% across simulations (2.5th–97.5th percentile, 43.1%–44.2%). After excluding temperature measurement-count features, XGBoost achieved an AUROC of 0.80 (95% CI 0.77–0.82) in UME and 0.77 (95% CI 0.76–0.78) in BWH. To assess model performance among patients with severe subsequent outcomes, we performed subgroup analyses stratified by subsequent ICU admission during the index hospital stay and mortality within 30 days after antibiotic initiation. Among 45 patients subsequently admitted to the ICU, 19 were classified as low risk for persistent fever, comprising 15 true negatives and 4 false negatives. For TabPFN, sensitivity was 0.84 (95% CI 0.68–0.96) in ICU patients compared with 0.68 (95% CI 0.63–0.72) in non-ICU patients, while specificity was 0.75 (95% CI 0.55–0.93) and 0.78 (95% CI 0.74–0.82), respectively. Discriminative performance was also similar, with an AUROC of 0.87 (95% CI 0.75–0.97) in ICU patients and 0.81 (95% CI 0.79–0.84) in non-ICU patients. By contrast, the naïve baseline model achieved a lower AUROC point estimate in the ICU subgroup (0.59, 95% CI 0.36–0.80) than in non-ICU patients (0.66, 95% CI 0.62–0.69). Among 67 patients who died within 30 days after antibiotic initiation, TabPFN achieved a sensitivity of 0.76 (95% CI 0.62–0.87), specificity of 0.74 (95% CI 0.57–0.86), and AUROC of 0.82 (95% CI 0.72–0.92).

### Analysis of clinical interventions

We retrospectively assessed the occurrence of antibiotic escalation and chest CT imaging performed between 36 and 96 h after antibiotic treatment initiation among patients correctly predicted not to develop fever. Among 365 hospital stays with antibiotic escalation in the UME cohort, 103 (28.2%; 95% CI 23.9–33.0%) were classified as true negatives. Similarly, among 223 hospital stays with chest CT imaging, 58 (26.0%; 95% CI 20.7–32.2%) were classified as true negatives (Supplementary Table 14).

## Discussion

In this multicenter retrospective study, we developed and externally validated machine learning (ML) models to predict persistent fever 48–72 h after initiation of antibiotic therapy in hospitalized cancer patients. Our findings demonstrate that short-term fever trajectories can be predicted with meaningful discrimination using routinely collected clinical variables, suggesting that such models may provide an additional signal during the critical reassessment window recommended by antimicrobial stewardship guidelines.

The models discriminated between patients who would and would not remain febrile, achieving an AUROC of 0.82 (95% CI, 0.79–0.84) in internal validation and 0.78 (95% CI, 0.77–0.79) in the external cohort. Thus, the model retained moderate discrimination at an independent academic center, although systematic overprediction, deviations of calibration slopes, and heterogeneity in sensitivity and specificity across temperature strata indicated differences in calibration and threshold-dependent performance between sites. The observed calibration shift also illustrates that decision thresholds derived in the development cohort should not be transferred unchanged across institutions, as differences in baseline risk and calibration may alter the clinical consequences of false-negative and false-positive predictions. Across demographic and clinical subgroups, performance was broadly consistent, while net-benefit profiles were similar across modeling approaches. These findings indicate that local model updating, potentially including adjustment of both the calibration intercept and slope, would be required before clinical deployment^13^.

In retrospective analyses, 28.2% (95% CI 23.9–33.0%) of antibiotic escalations and 26.0% (95% CI 20.7–32.2%) of chest CT scans occurred in patients correctly predicted not to develop persistent fever. These findings represent descriptive overlap and should not be interpreted as indicating that these interventions were unnecessary or could safely have been withheld. Fever persistence is a proxy marker of short-term clinical trajectory rather than treatment failure, while fever resolution does not exclude ongoing infection or other indications for escalation or imaging. Moreover, interventions during the outcome window may themselves influence subsequent fever trajectories, precluding causal or counterfactual interpretation of these associations. Nonetheless, they illustrate how fever-persistence predictions could provide additional context for clinical judgment and motivate prospective evaluation of their influence on decision-making.

Among the strengths of our study are the inclusion of two independent tertiary cancer centers with differing oncologic case mixes and fever prevalence. Second, we evaluated a wide spectrum of ML approaches, reducing dependence on a single algorithmic paradigm. Third, SHAP-based interpretability analyses showed broadly consistent feature rankings between datasets for individual models. While agreement across model families was more limited, temperature-derived variables consistently remained among the highest-ranked features associated with model predictions. Fourth, standardized data harmonization and a common feature-engineering pipeline facilitated external application across institutions. Finally, the incorporation of a time-series forecasting procedure demonstrated the feasibility of integrating longitudinal forecasting with static classification for short-term clinical prediction. Several limitations must be acknowledged. Although discrimination was retained at a second center, our study included only two high-resource academic hospitals, and generalizability to community hospitals, low-resource settings, or institutions with different documentation practices remains uncertain. The retrospective design also precludes conclusions regarding real-world clinical utility or safety. Determination of the primary outcome required at least one documented body temperature measurement during the 48–72-h outcome window, restricting the analysis to patients remaining under active clinical observation. Because absent measurements may be associated with discharge, ICU transfer, death, or changes in monitoring intensity, outcome availability may have introduced selection and verification bias.

While sensitivity analyses suggested only a modest effect of differential monitoring intensity on fever ascertainment and little dependence of model discrimination on measurement-count features in our datasets, residual surveillance-intensity bias may remain because the probability of detecting at least one febrile measurement is influenced by the number and timing of temperature observations, while monitoring patterns may themselves reflect clinician concern, patient condition, and site-specific workflows.

Beyond differences in measurement frequency, body temperatures were obtained during routine clinical care rather than according to a standardized study-specific measurement protocol. Routine bedside temperature measurements may be affected by measurement technique, device- and site-related variation, and recording errors, introducing additional measurement variability into retrospectively collected EHR data. Previous work has demonstrated that a proportion of implausible bedside temperature recordings can result from the measurement or documentation process rather than true physiological values^14^. Accordingly, because temperature-derived variables contributed substantially to model predictions and fever status was itself defined using a fixed temperature threshold, measurement variability could affect both predictor values and outcome classification. Prospective validation using standardized temperature measurement procedures would therefore be valuable.

False-negative predictions remain particularly consequential in safety-critical settings. Although false negatives were less frequent among patients subsequently requiring ICU care, incremental net benefit over a treat-all strategy was small at lower, safety-oriented thresholds. Youden’s J was therefore used only as a balanced reference threshold rather than a proposed clinical deployment threshold. Higher-sensitivity operating points reduced false-negative rates and modestly increased NPV but substantially decreased specificity and the proportion classified as low risk. Clinical threshold selection should therefore reflect the relative consequences of false-negative and false-positive predictions rather than statistical optimization alone. Accordingly, the present results do not establish a threshold at which persistent fever can be safely ruled out for the purpose of antibiotic de-escalation, withholding diagnostic imaging, or other treatment decisions, and model predictions should not be used in isolation for such decisions. The forecast-based feature may additionally limit applicability in simple point-of-care tools because it requires longitudinal data and a time-series modeling step. Likewise, while forward-fill imputation was chosen to represent the most recently available clinical information, it may not be the statistically optimal approach. Finally, because patients were included irrespective of neutropenia status, performance was not specifically evaluated in patients with neutropenia, representing an important population for future investigation.

Our findings are broadly consistent with prior literature demonstrating that ML applied to structured EHR data can achieve moderate to high discrimination for short-term outcomes in infectious diseases and critical care^15,16^. As in our study, more complex model architectures have often provided incremental rather than dramatic gains over simpler approaches such as logistic regression^17^. Similar to sepsis prediction studies^18^, dynamic vital signs, particularly temperature, carried much of the predictive signal, supporting the value of longitudinal trajectories for individualized risk estimation.

Several avenues for further research follow from this work. Prospective evaluation is needed to determine the safety and acceptability of ML-assisted fever prediction in clinical practice. Initial evaluation could combine silent-mode deployment with counterfactual simulation of decision pathways to estimate potential effects on CT use and antimicrobial escalation without altering patient care^15,19,20^. Such studies should specifically examine calibration, false-negative predictions, and clinically relevant downstream outcomes at prespecified operating thresholds before model-informed changes to antimicrobial or diagnostic management are considered. Studies across settings should further assess workflow integration, clinician acceptance, and governance. Incorporating information from free-text clinical notes and pathogen-specific data may improve model accuracy and enable more specific downstream tasks, including identification of patients who may benefit from diagnostic imaging.

In summary, ML models predicted persistent fever in hospitalized cancer patients receiving broad-spectrum antibiotics with moderate discrimination at both the development and external validation sites. Dynamic temperature-derived features carried most of the predictive signal, suggesting that longitudinal fever patterns provide information beyond individual temperature measurements. The models should be viewed as adjunctive risk estimates rather than treatment recommendations and should not be used as standalone rule-out tools for antimicrobial or diagnostic decision-making. Clinical implementation would require local calibration, context-specific threshold selection, and prospective validation specifically evaluating safety at the intended clinical operating point.

## Methods

### Data sources

The dataset used to train and internally validate the prediction models was extracted from the existing HL7® FHIR® (Fast Healthcare Interoperability Resources) infrastructure of University Hospital Essen. This database combines all relevant patient data from the different primary databases in a unified and standardized data structure. It is comprised of all patient visits from the University Medicine Essen (UME) holding, totaling more than 3.0 million distinct patient visits^21^.

For the training cohort, completed hospital stays between 2020-01 and 2025-03 were used. The external validation to assess the models’ transportability to a different region and clinical setting was conducted with data from the Brigham and Women’s Hospital (BWH) in Boston, MA, recorded between 2015-06 and 2024-09 and extracted from the hospital’s institutional EPIC system.

Variables were mapped to common clinical definitions and time windows, with measurement units converted where necessary to UME units. Antibiotic exposures were harmonized using ATC-based medication categories. The same feature-engineering and preprocessing pipeline was subsequently applied to both cohorts.

Measurement or documentation errors were excluded from the dataset, such as implausible values or duplicate recordings. Missing values in longitudinal variables were forward-filled within the same hospital stay to represent the most recently available clinical information at each time point in routine care. Where no forward-filling was available, reference values were used. Data preprocessing steps are detailed in Supplementary Table 15, missingness and reference values are reported in Supplementary Tables 16–17.

### Study cohort

The study cohort included hospital stays of patients aged ≥18 years with hematological or oncological admission diagnoses (ICD-10 codes C00-C96). We further restricted to patients with fever during 24 to 48 h after the first administration of broad-spectrum IV antibiotics. Fever was identified by at least one body temperature measurement ≥ 38.0 °C, and ≥ 38.3 °C for the sensitivity analyses^22^. Patients who received antimicrobial prophylaxis only were excluded. Neutropenia was not an inclusion criterion, and patients were included irrespective of their leukocyte or neutrophil count. Furthermore, at least one body temperature measurement had to be recorded 48 to 72 h after the first documented administration of antibiotics.

### Covariates

A total of 44 routinely collected variables were extracted based on prior literature^23^ and medical expert opinion: admission diagnosis grouped into leukemia, other hematological malignancies or solid tumors, 30 comorbidity categories derived from previously recorded ICD-10 codes for constructing the Elixhauser comorbidity score^24^, age at admission, sex and the length of hospital stays from admission until the first administration of antibiotics. In addition, longitudinal measurements of laboratory values and vital parameters were included: Leukocytes, serum creatinine, C-reactive Protein (CrP), hemoglobin, bilirubin, body temperature, mean arterial blood pressure, heart rate, and peripheral oxygen saturation.

Body temperature measurements were extracted from routinely documented vital signs in the respective institutional electronic health records. Temperatures were measured using digital thermometers and standard clinical measurement methods, with measurements obtained predominantly non-invasively, e.g., via in-ear thermometry. Temperature values recorded in Fahrenheit in the BWH cohort were converted using °C = (°F − 32) × 5/9. Measurement frequency was not standardized for the main analysis and therefore reflects local monitoring practices at each institution.

Model features included maximum values of laboratory and vital-sign measurements within 0–24 and 24–48 h after antibiotic initiation. Temperature features additionally comprised maximum and mean values in these windows, the last value before the 48-h prediction cutoff, the proportion of measurements ≥38.0 °C, measurement frequency, variability, first-to-last change, range, time-of-day maxima, and overall 0–48-h trend, skewness, and Fourier magnitude. Using a time series foundation model, the maximum forecasted temperature for 48–72 h was included as an additional predictor.

Categorical variables were one-hot encoded, and continuous covariates were normalized using standard scaling. In total, 52 engineered features were derived from the initial covariates (Supplementary Table 18).

### Model development

In the model development phase, we first explored a broad range of candidate algorithms to determine an appropriate modeling approach for this prediction task. Initial prototyping included deep learning architectures for tabular prediction^25,26^ and time-series classification^27,28^, as well as the large language model Llama 3.3-70B using in-context learning^29^. During preliminary experimentation, these approaches did not demonstrate performance advantages over more conventional tabular classification models, while requiring substantially more computational resources.

Furthermore, we conducted an exploratory analysis to assess the impact of incorporating potentially relevant variables not routinely available in structured EHR data. Specifically, we extracted information on the potential infection focus, antimicrobial resistance, overall illness, early clinical trajectory, microbiologic factors and host vulnerability from unstructured clinical notes in the UME cohort using the large language model Qwen3-Next-80B-A3B-Instruct^30^, after excluding content recorded within or after the prediction window to prevent data leakage. However, these features did not improve model performance, were not available in a complete or standardized form in the clinical notes and were also not available in the external validation cohort. Detailed extraction methods and results are provided in Supplementary Tables 19–22.

Consequently, the main analysis focused on gradient boosting models (XGBoost^31^, CatBoost^32^), Random Forest^33^ as well as logistic regression tuned to explore L1-regularized (lasso), L2-regularized (Ridge), and Elastic net penalties^34^. As a deep learning architecture that shows strong performance on smaller tabular datasets with less than 10,000 rows, the generative transformer-based foundation model TabPFN was included^35^. For baseline comparison, we included an unregularized logistic regression model, equivalent to a generalized linear model (GLM) with a binomial distribution and logit link^36^. Additionally, a naïve baseline predictor using only the most recent body temperature measured at 48 hours after initiation of antibiotic therapy was included to approximate clinical decision-making based on the latest available measurement. All models were trained and evaluated using identical development procedures, predictor sets, and outcome definitions. Nested cross-validation was grouped by patient identifier to ensure that all admissions from the same patient remained within the same fold in both the inner and outer loops. Confidence intervals were estimated using patient-level cluster bootstrapping.

To capture short-term temporal dynamics relevant to fever evolution, we incorporated a forecast-derived feature using a time series model. Integrating this feature into the downstream classification model represents a feature-level multimodal approach^37^, complementing structured clinical variables without imposing additional data requirements. For each hospital stay, all body temperature measurements from hospital admission up to 48 h after initiation of antibiotic therapy were used as input. Based on these data, the model forecasted future temperature values between 48 and 72 h after treatment initiation. The maximum forecasted temperature within this interval was then included as an additional predictor in the classification model. Importantly, to effectively avoid data leakage, time series of body temperature measurements were first split in context (hospital admission up to 48 h after start of antibiotic therapy) and prediction windows (48–72 h after treatment initiation). Only data from the context period were used to inform the time series model. Temperature measurements were resampled to 8-h intervals to ensure a consistent temporal structure for the model input.

For the forecasting component, we used the foundation model Chronos-2, which has shown strong zero-shot performance on short time series and does not require fine-tuning^38^. This approach enabled efficient integration of short-term and irregular temporal dynamics into the feature engineering pipeline without additional model training or data requirements. Detailed implementation and inference settings for the Chronos-2 forecasting component, including context lengths and handling of missing intervals, are provided in Supplementary Table 23. Hyperparameters of classification models were determined using Bayesian optimization. Detailed search spaces for each model are described in Supplementary Table 24.

This study was implemented using Python 3.11, with the primary libraries for model development including Scikit-learn (v1.1.3) and Optuna (v2.10.1). ChatGPT 5.1 and Gemini Flash were used to perform code consistency as well as spelling and grammatical checks.

Methods and results were reported in accordance with the TRIPOD + AI guideline^39^ (Supplementary File 1).

### Model evaluation

We evaluated model performance on the training dataset using a nested 5-fold cross-validation (CV) over 20 trials. Hyperparameter optimization was performed in the inner loop to prevent data leakage into the outer loop, where each split was trained with the optimized parameters^40^. All data-dependent preprocessing steps were fitted exclusively on the training data within each cross-validation fold and subsequently applied unchanged to the corresponding validation data. No information from the outer validation folds was used to estimate preprocessing parameters. For external validation, preprocessing parameters estimated from the UME development cohort were applied unchanged to the BWH cohort. Hyperparameters were re-optimized on the complete UME development cohort using the same predefined search space and 5-fold cross-validation in inner-loop tuning. The configuration with the highest mean AUROC was then used to fit the final model on the full UME cohort before evaluation on BWH. Probability calibration was performed using Platt scaling based exclusively on training data within the cross-validation framework.

To obtain robust estimates of uncertainty, all performance metrics were calculated using nonparametric bootstrapping to derive 95% confidence intervals. Discrimination was assessed using the area under the receiver operating characteristic curve (AUROC) and the area under the precision–recall curve (AUPRC). Calibration was evaluated using calibration-in-the-large, calibration slope and Brier score. Threshold-dependent metrics included sensitivity, specificity, positive predictive value (PPV), negative predictive value (NPV), F1-score, and accuracy. Classification thresholds were derived using Youden’s J statistic in the internal dataset and applied unchanged to the external cohort to assess how population-specific calibration shifts affect diagnostic performance, simulating real-world deployment of a locked model. In addition, we evaluated safety-oriented operating points corresponding to sensitivities of ≥90%, ≥95%, and ≥98% in the internal cohort and applied resulting thresholds unchanged to the external cohort.

Experiments on the UME dataset evaluated the impact of incorporating the forecast-derived feature by comparing model performance with and without this variable. In addition, the effect of using a dichotomized version of the forecasted temperature from the Chronos-2 model was assessed.

Decision curve analysis (DCA) was performed to evaluate threshold-dependent utility for identifying patients at increased risk of persistent fever. In this context, “treat” denotes classification as high risk rather than a specific clinical intervention. The threshold probability determines the relative weighting of false-positive and false-negative classifications. The evaluated thresholds of 0.20–0.60 were exploratory and were not intended to represent intervention-specific thresholds.

Forecast performance was evaluated using mean absolute error (MAE), mean squared error (MSE), and symmetric mean absolute percentage error (sMAPE). To contextualize Chronos-2 performance, forecasts were additionally compared with a seasonal-naïve approach and AutoARIMA using the same input and prediction windows.

Feature importance was assessed using SHAP (SHapley Additive exPlanations) values, which quantify the contribution of each feature to individual model predictions^41^. SHAP magnitudes were not compared directly between models. Cross-model and cross-dataset agreement was assessed using Spearman rank correlations of feature rankings and Jaccard indices of top-ranked feature overlap.

To evaluate model performance across clinically relevant subgroups, we examined differences in predicted outcomes within predefined patient strata. Continuous variables were categorized to facilitate subgroup comparisons. Sensitivity analyses were performed using an alternative fever threshold of ≥ 38.3 °C to assess robustness to outcome definition. To evaluate the potentially harmful impact of false-negative predictions, we compared the proportion of missed fever cases between patients who were subsequently admitted to the intensive care unit (ICU) and those who were not, and additionally assessed model performance among patients who died within 30 days after antibiotic initiation. To explore the effect of differential temperature-monitoring intensity on fever prevalence, BWH temperature measurements during the 48–72-h outcome window were downsampled over 1000 iterations to match the empirical UME measurement-count distribution, and fever prevalence was recalculated for each iteration. Additionally, XGBoost was retrained after excluding temperature measurement-count signals. To further assess the contribution of temperature-derived information, additional analyses included a logistic-regression model using standard temperature summaries and XGBoost models restricted to either a temperature-derived forecast or non-temperature predictors, using the same validation framework as the primary analyses.

To explore how model predictions relate to subsequent clinical management, we retrospectively assessed antibiotic escalation (definition provided in Supplementary Table 25) and chest CT imaging performed between 36 and 96 h after treatment initiation among patients correctly predicted not to develop persistent fever. This interval was used to characterize intervention patterns surrounding the clinical reassessment period and was not intended to represent prospective model-guided decision-making. Confidence intervals for intervention proportions were estimated using patient-level cluster bootstrapping, with unique patients resampled with replacement and all corresponding hospital stays retained within each bootstrap sample. For the intervention analysis, model classifications were based exclusively on out-of-fold predictions from the outer cross-validation folds, ensuring that each hospital stay was evaluated by a model that had not been trained on that observation.

### Ethical approval

This study was conducted in accordance with the principles of the Declaration of Helsinki. A waiver of consent was obtained from the Medical Ethics Committee of the University Duisburg-Essen (reference number 23-11573-BO), due to the retrospective nature of the study and the use of anonymized data from existing medical records, with no impact on patient care. The Institutional Review Board of Mass General Brigham approved of this study (reference number 2017P000682).

## Supplementary information

Supplementary information

## Data Availability

In concordance with German legal code, the data underlying this study cannot be shared publicly, however, we will share a pseudo-anonymized dataset upon reasonable request with researchers for validation purposes on an individual basis. For original data, please contact Christopher.Sauer@UK-Essen.de.

## Code availability

The Python code used to perform analyses is available from the following public GitHub repository: https://github.com/gernotpuc/ml-fever-prediction.
