# Supplementary material for: Predicting persistent fever in patients with cancer receiving antibiotics: a machine learning approach
Source: NPJ Digit Med. 2026 Oct 31;9:830. doi: 10.1038/s41746-026-03449-w (PMC13723596; doi:10.1038/s41746-026-03449-w)
Supplement: Supplementary file 1 [file 41746_2026_3449_MOESM1_ESM.docx]

Supplementary Materials

Supplement to: Pucher G., Kanjilal S., Kopp K., et al. (2026): Predicting Persistent Fever in Patients with Cancer Receiving Antibiotics: A Machine Learning Approach.

Table of contents

[Supplementary Table 1. Distribution of hospital stays per patient and cohort. UME = University Medicine Essen; BWH = Brigham and Women's Hospital.	4](#_Toc241777372)

[Supplementary Table 2. Clinical outcomes and documentation patterns of body temperature measurements of UME patients excluded from the final cohort because of missing body temperature measurements during the outcome window Within 48 to 72 hours after start of antibiotics, compared to the final UME cohort. UME = University Medicine Essen.	4](#_Toc241777373)

[Supplementary Table 3. Distribution of maximum body temperature across 0–24 h, 24–48 h, and 48–72 h assessment windows, stratified by persistent fever status. UME = University Medicine Essen; BWH = Brigham and Women’s Hospital; AB = antibiotics. Tmax = highest measured body temperature.	4](#_Toc241777374)

[Supplementary Table 4. Comparison of AUC of models with and without using the forecasted fever value from the Chronos-2 forecasting model, based on 5-fold nested CV on the UME dataset. UME= University Medicine Essen; GLM = Generalized linear model; LR = Regularized logistic regression; RF = Random Forest; XGB = XGBoost; CB = CatBoost; TPFN = TabPFN.	5](#_Toc241777375)

[Supplementary Table 5. Performance metric per model during 5-fold nested CV over 20 trials on the UME dataset. UME = University Medicine Essen; GLM = Generalized linear model; LR = Regularized logistic regression; RF = Random Forest; XGB = XGBoost; CB = CatBoost; TPFN = TabPFN.	5](#_Toc241777376)

[Supplementary Table 6. Performance metric per model during external validation on the BWH dataset. BWH = Brigham and Women’s Hospital; GLM = Generalized linear model; LR = Regularized logistic regression; RF = Random Forest; XGB = XGBoost; CB = CatBoost; TPFN = TabPFN.	6](#_Toc241777377)

[Supplementary Table 7. Regression metrics of Chronos-2 based forecasted body measurements (°C) 48-72 hours after start of antibiotic therapy, resample to 8h intervals. MAE = Mean absolute error; MSE = Mean square error; SMAPE = Symmetric mean absolute percentage error.	7](#_Toc241777378)

[Supplementary Table 8. Top 10 features with highest SHAP values per model and dataset. Rank agreement through mean Spearman rank correlation and Overlap through Jaccard Index, per dataset and across models and per model and across dataset; For feature descriptions, see Supplementary Table 17. UME = University Medicine Essen; BWH = Brigham and Women’s Hospital; GLM = Generalized linear model; LR = Regularized logistic regression; RF = Random Forest; XGB = XGBoost; CB = CatBoost; TPFN = TabPFN.	7](#_Toc241777379)

[Supplementary Table 9. Top 10 features with highest Permutation Feature Importance values for the XGBoost model and per dataset. For feature descriptions, see Supplementary Table 17. UME = University Medicine Essen; BWH = Brigham and Women’s Hospital; XGB = XGBoost.	9](#_Toc241777380)

[Supplementary Table 10. Performance of temperature-focused comparator and feature-ablation models. UME = University Medicine Essen; BWH = Brigham and Women’s Hospital; XGB = XGBoost; LR = Logistic Regression.	9](#_Toc241777381)

[Supplementary Table 11. Stratified discriminative performance per model during 5-fold nested CV over 20 trials on the UME dataset. UME = University Medicine Essen; GLM = Generalized linear model; LR = Regularized logistic regression; RF = Random Forest; XGB = XGBoost; CB = CatBoost; TPFN = TabPFN.	9](#_Toc241777382)

[Supplementary Table 12. Stratified discriminative performance per model during external validation on the BWH dataset. BWH = Brigham and Women’s Hospital; GLM = Generalized linear model; LR = Regularized logistic regression; RF = Random Forest; XGB = XGBoost; CB = CatBoost; TPFN = TabPFN.	10](#_Toc241777383)

[Supplementary Table 13. Discriminative performance per model during 5-fold nested CV over 20 trials on the UME dataset and external validation on the BWH dataset under an adapted fever definition threshold of $\geq$38.3 °C. UME = University Medicine Essen; BWH = Brigham and Women’s Hospital; GLM = Generalized linear model; LR = Regularized logistic regression; RF = Random Forest; XGB = XGBoost; CB = CatBoost; TPFN = TabPFN.	10](#_Toc241777384)

[Supplementary Table 14. Descriptive overlap between true-negative model predictions and antibiotic escalation or chest CT imaging for the UME cohort. UME = University Medicine Essen.	10](#_Toc241777385)

[Supplementary Table 15. Data preprocessing steps.	11](#_Toc241777386)

[Supplementary Table 16. Missing and forward-filled values per engineered feature and cohort. UME = University Medicine Essen. BWH = Brigham and Women’s hospital.	12](#_Toc241777387)

[Supplementary Table 17. Reference values used where forward-filling was not available.	13](#_Toc241777388)

[Supplementary Table 18. Engineered features derived from the initial covariates.	13](#_Toc241777389)

[Supplementary Table 19. Settings of Qwen3-Next-80B-A3B-Instruct for feature extraction from clinical notes.	15](#_Toc241777390)

[Supplementary Table 20. Results from repeated-inference stability testing for LLM-based feature extraction across 5 runs.	15](#_Toc241777391)

[Supplementary Table 21. Prompt template for LLM-based clinical phenotype extraction.	16](#_Toc241777392)

[Supplementary Table 22. Agreement between LLM-generated features and a clinician reference standard from 30 clinical notes.	17](#_Toc241777393)

[Supplementary Table 23. Implementation settings of the Chronos-2 forecasting model.	17](#_Toc241777394)

[Supplementary Table 24. Hyperparameter search spaces for utilized machine learning models.	18](#_Toc241777395)

[Supplementary Table 25. Definition of antibiotic escalation strategies used for evaluation.	18](#_Toc241777396)

[Supplementary File 1. Tripod+AI Checklist.	20](#_Toc239700093)

Supplementary Table 1. Distribution of hospital stays per patient and cohort. UME = University Medicine Essen; BWH = Brigham and Women's Hospital.

|  | UME cohort | | BWH cohort | |
| --- | --- | --- | --- | --- |
| Number of hospital stays per patient, n | Unique Patients, n | Patients, % | Unique Patients, n | Patients, % |
| 1 | 642 | 80.86 | 5412 | 86.51 |
| 2 | 121 | 15.24 | 633 | 10.12 |
| 3 | 22 | 2.77 | 141 | 2.25 |
| 4 | 8 | 1.01 | 49 | 0.78 |
| 5 | 1 | 0.13 | 15 | 0.24 |
| 6 | 0 | 0.00 | 5 | 0.08 |
| 7 | 0 | 0.00 | 1 | 0.02 |

Supplementary Table 2. Clinical outcomes and documentation patterns of body temperature measurements of UME patients excluded from the final cohort because of missing body temperature measurements during the outcome window Within 48 to 72 hours after start of antibiotics, compared to the final UME cohort. UME = University Medicine Essen.

| Clinical outcomes | Within 48 to 72 hours after start of antibiotics | |
| --- | --- | --- |
|  | Hospital stays excluded because of missing body temperature in the prediction window, UME, n=244 | Hospital stays included in the final cohort, UME, n=987 |
| Death, n | 8 | 7 |
| Discharge, n | 44 | 49 |
| Transfer to ICU, n | 32 | 37 |
| Transfer to other ward, n | 28 | 41 |
| Documentation patterns of body temperature measurements | Hospital stays excluded because of missing body temperature in the prediction window, UME, n=244 | Hospital stays included in the final cohort, UME, n=987 |
| Temporary monitoring or documentation gap during the prediction window, with at least one body temperature available at any time after the prediction window | 68 | 0 |
| No ongoing monitoring or documentation of body temperature | 64 | 0 |

Supplementary Table 3. Distribution of maximum body temperature across 0–24 h, 24–48 h, and 48–72 h assessment windows, stratified by persistent fever status. UME = University Medicine Essen; BWH = Brigham and Women’s Hospital; AB = antibiotics. Tmax = highest measured body temperature.

| Cohort | Persistent fever 48-72 h | Window after first AB | Mean (SD), °C | Min-Max, °C | 5th-95th percentile, °C | 1st-99th percentile, °C | Tmax ≥38.0°C, n (%) |
| --- | --- | --- | --- | --- | --- | --- | --- |
| UME | Yes | 0–24 h | 38.7 (0.7) | 36.2-40.8 | 37.6-39.9 | 37.0-40.4 | 476/533 (89.3%) |
| UME | No | 0–24 h | 38.4 (0.8) | 36.0-40.9 | 37.0-39.6 | 36.6-40.1 | 350/454 (77.1%) |
| UME | Yes | 24–48 h | 38.8 (0.6) | 38.0-40.8 | 38.0-39.8 | 38.0-40.5 | 533/533 (100.0%) |
| UME | No | 24–48 h | 38.4 (0.4) | 38.0-40.7 | 38.0-39.2 | 38.0-39.8 | 454/454 (100.0%) |
| UME | Yes | 48–72 h | 38.7 (0.6) | 38.0-40.9 | 38.0-39.8 | 38.0-40.3 | 533/533 (100.0%) |
| UME | No | 48–72 h | 37.2 (0.5) | 35.8-37.9 | 36.4-37.9 | 36.0-37.9 | 0/454 (0.0%) |
| BWH | Yes | 0–24 h | 38.6 (0.8) | 36.3-43.2 | 37.2-39.8 | 36.8-40.4 | 2608/3442 (75.8%) |
| BWH | No | 0–24 h | 38.3 (0.8) | 36.4-43.1 | 37.1-39.6 | 36.8-40.2 | 2467/3967 (62.2%) |
| BWH | Yes | 24–48 h | 38.8 (0.6) | 38.0-43.2 | 38.1-39.7 | 38.0-40.3 | 3442/3442 (100.0%) |
| BWH | No | 24–48 h | 38.5 (0.5) | 38.0-42.7 | 38.0-39.4 | 38.0-39.7 | 3967/3967 (100.0%) |
| BWH | Yes | 48–72 h | 38.7 (0.6) | 38.0-42.7 | 38.0-39.1 | 38.0-40.2 | 3442/3442 (100.0%) |
| BWH | No | 48–72 h | 37.4 (0.4) | 35.8-37.9 | 36.8-37.9 | 36.5-37.9 | 0/3967 (0.0%) |

Supplementary Table 4. Comparison of AUC of models with and without using the forecasted fever value from the Chronos-2 forecasting model, based on 5-fold nested CV on the UME dataset. UME= University Medicine Essen; GLM = Generalized linear model; LR = Regularized logistic regression; RF = Random Forest; XGB = XGBoost; CB = CatBoost; TPFN = TabPFN.

| **Model** | **AUC_95% CI_** |
| --- | --- |
| GLM | 0.72 (CI 0.69-0.74) |
| GLM+Chronos-2 | 0.73 (CI 0.70-0.76) |
| LR | 0.73 (CI 0.70-0.76) |
| LR+Chronos-2 | 0.76 (CI 0.73-0.78) |
| RF | 0.74 (CI 0.71-0.77) |
| RF+Chronos-2 | 0.76 (CI 0.74-0.79) |
| CB | 0.77 (CI 0.74-0.80) |
| CB+Chronos-2 | 0.79 (CI 0.76-0.82) |
| XGB | 0.77 (CI 0.74-0.80) |
| XGB+Chronos-2 | 0.80 (CI 0.77-0.82) |
| TPFN | 0.79 (CI 0.76-0.81) |
| TPFN+Chronos-2 | 0.82 (CI 0.79-0.84) |
| Chronos-2 | 0.74 (CI 0.71-0.77) |

Supplementary Table 5. Performance metric per model during 5-fold nested CV over 20 trials on the UME dataset. UME = University Medicine Essen; GLM = Generalized linear model; LR = Regularized logistic regression; RF = Random Forest; XGB = XGBoost; CB = CatBoost; TPFN = TabPFN.

| Metric | Naive | GLM | LR | RF | XGB | CB | TPFN |
| --- | --- | --- | --- | --- | --- | --- | --- |
| **Discrimination** |  |  |  |  |  |  |  |
| AUROC (95% CI) | 0.66 (0.62-0.69) | 0.73 (0.70-0.76) | 0.76 (0.73-0.78) | 0.76 (0.74-0.79) | 0.80 (0.77-0.82) | 0.79 (0.76-0.82) | 0.82 (0.79-  0.84) |
| AUPRC (95% CI) | 0.68 (0.64-0.73) | 0.76 (0.72-0.80) | 0.79 (0.75-0.82) | 0.78 (0.74-0.81) | 0.81 (0.77-0.84) | 0.81 (0.78-0.85) | 0.84 (0.81-0.87) |
| **Calibration** |  |  |  |  |  |  |  |
| Calibration intercept (95% CI) | 0.50 (0.34-0.66) | 0.14 (0.00-0.27) | 0.17 (0.03-0.30) | -0.01  (-0.16-0.13) | 0.00 (-0.15-0.15) | -0.05  (-0.20-0.09) | -0.08 (-0.23-0.07) |
| Calibration slope (95% CI) | 0.86 (0.65-1.08) | 0.69 (0.58-0.82) | 1.23 (1.04-1.45) | 0.86 (0.74-0.99) | 1.02 (0.90-1.17) | 0.97  (0.85-1.10) | 0.96 (0.86-1.08) |
| Brier score (95% CI) | 0.25 (0.24-0.26) | 0.21 (CI 0.20- 0.23) | 0.20 (0.19-0.21) | 0.20 (0.19-0.21) | 0.18 (0.17-0.20) | 0.19 (0.17-0.20) | 0.17 (0.16-0.18) |
| **Threshold-based metrics** |  |  |  |  |  |  |  |
| Sensitivity (95% CI) | 0.37 (0.33-0.41) | 0.63 (0.59-0.67) | 0.69 (0.65-0.72) | 0.66 (0.62-0.70) | 0.70 (0.66-0.73) | 0.70 (0.66-0.73) | 0.70 (0.65-0.73) |
| Specificity (95% CI) | 0.81  (0.78-0.85) | 0.70 (0.66-0.74) | 0.74 (0.70-0.78) | 0.71 (0.67-0.75) | 0.75 (0.71-0.79) | 0.76 (0.72-0.80) | 0.78 (0.74-0.81) |
| PPV (95% CI) | 0.70 (0.65-0.75) | 0.72 (0.68-0.76) | 0.76 (0.72-0.80) | 0.73 (0.69-0.77) | 0.76 (0.73-0.80) | 0.77 (0.73-0.81) | 0.79 (0.74-0.82) |
| NPV (95% CI) | 0.52 (0.49-0.56) | 0.63 (0.59-0.67) | 0.67 (0.63-0.71) | 0.64 (0.60-0.68) | 0.68 (0.64-0.72) | 0.68 (0.64-0.72) | 0.68 (0.64-0.72) |
| F1 score (95% CI) | 0.49 (0.44-0.53) | 0.68 (0.65-0.71) | 0.72 (0.69-0.75) | 0.69 (0.66-0.72) | 0.73 (0.70-0.76) | 0.73 (0.70-0.76) | 0.74 (0.70-0.77) |
| Accuracy (95% CI) | 0.63 (0.62-0.64) | 0.67 (0.64-0.70) | 0.71 (0.68-0.74) | 0.68 (0.65-0.71) | 0.72 (0.69-0.75) | 0.73 (0.70-0.75) | 0.74 (0.71-0.77) |

Supplementary Table 6. Performance metric per model during external validation on the BWH dataset. BWH = Brigham and Women’s Hospital; GLM = Generalized linear model; LR = Regularized logistic regression; RF = Random Forest; XGB = XGBoost; CB = CatBoost; TPFN = TabPFN.

| Metric | Naive | GLM | LR | RF | XGB | CB | TPFN |
| --- | --- | --- | --- | --- | --- | --- | --- |
| **Discrimination** |  |  |  |  |  |  |  |
| AUROC (95% CI) | 0.69 (0.68-0.70) | 0.71 (0.70-0.72) | 0.75 (0.74-0.76) | 0.75 (0.74-0.76) | 0.77 (0.76-0.78) | 0.77 (0.76-0.78) | 0.78 (0.77-  0.79) |
| AUPRC (95% CI) | 0.21 (CI 0.19-0.22) | 0.70 (0.68-0.72) | 0.72 (0.70-0.73) | 0.73 (0.71-0.74) | 0.76 (0.74-0.77) | 0.75 (0.74-0.77) | 0.76 (0.75-0.78) |
| **Calibration** |  |  |  |  |  |  |  |
| Calibration intercept (95% CI) | 3.28 (CI 2.99 – 3.55) | 0.20 (0.14-0.26) | -0.17 (-0.21 to -0.11) | -0.42  (-0.47 to -0.36) | -0.41 (-0.46 to -0.36) | -0.47  (-0.51 to -0.41) | -0.48 (-0.53 to -0.42) |
| Calibration slope (95% CI) | 1.90 (CI 1.75 – 2.06) | 0.34 (0.29-0.38) | 1.37 (1.23-1.49) | 0.86 (0.74-0.99) | 1.18 (1.12-1.24) | 1.26  (1.20-1.33) | 1.17 (1.11-1.23) |
| Brier score (95% CI) | 0.34 (CI 0.33 – 0.34) | 0.23 (CI 0.23- 0.24) | 0.21 (0.20-0.21) | 0.21 (0.21-0.22) | 0.20 (0.19-0.20) | 0.20 (0.20-0.21) | 0.20 (0.19-0.20) |
| **Threshold-based metrics** |  |  |  |  |  |  |  |
| Sensitivity (95% CI) | 0.33 (0.31-0.34) | 0.56 (0.54-0.57) | 0.66 (0.65-0.68) | 0.66 (0.64-0.67) | 0.68 (0.67-0.70) | 0.69 (0.67-0.70) | 0.69 (0.67-0.70) |
| Specificity (95% CI) | 0.88 (0.87-0.89) | 0.78 (0.77-0.79) | 0.72 (0.70-0.73) | 0.72 (0.70-0.73) | 0.74 (0.73-0.76) | 0.74 (0.73-0.76) | 0.76 (0.74-0.77) |
| PPV (95% CI) | 0.71 (0.69-0.73) | 0.69 (0.67-0.70) | 0.67 (0.66-0.69) | 0.67 (0.65-0.69) | 0.70 (0.68-0.71) | 0.70 (0.68-0.71) | 0.70 (0.69-0.72) |
| NPV (95% CI) | 0.60 (0.59-0.62) | 0.67 (0.66-0.68) | 0.71 (0.70-0.73) | 0.71 (0.69-0.72) | 0.73 (0.71-0.74) | 0.73 (0.72-0.74) | 0.73 (0.71-0.74) |
| F1 score (95% CI) | 0.45 (0.43-0.47) | 0.62 (0.60-0.63) | 0.67 (0.66-0.68) | 0.66 (0.65-0.68) | 0.69 (0.67-0.70) | 0.69 (0.68-0.70) | 0.70 (0.68-0.71) |
| Accuracy (95% CI) | 0.63 (0.62-0.64) | 0.68 (0.67-0.69) | 0.69 (0.68-0.70) | 0.69 (0.68-0.70) | 0.71 (0.70-0.72) | 0.71 (0.70-0.72) | 0.72 (0.71-0.73) |

Supplementary Table 7. Regression metrics of Chronos-2 based forecasted body measurements (°C) 48-72 hours after start of antibiotic therapy, resample to 8h intervals. MAE = Mean absolute error; MSE = Mean square error; SMAPE = Symmetric mean absolute percentage error.

| Metric | University Medicine Essen | | |
| --- | --- | --- | --- |
|  | Chronos-2 | Seasonal-Naive | AutoARIMA |
| MAE (95% CI) | 0.78 (0.76-0.81) | 0.87 (0.85-0.89) | 0.83 (0.81-0.85) |
| MSE (95% CI) | 0.97 (0.92-1.02) | 1.18 (1.13-1.23) | 1.07 (1.02-1.13) |
| SMAPE (95% CI) | 1.04 (1.01-1.07) | 1.21 (1.18-1.24) | 1.10 (1.07-1.13) |
|  | Brigham and Women’s Hospital | | |
|  | Chronos-2 | Seasonal-Naive | AutoARIMA |
| MAE (95% CI) | 0.72 (0.71-0.73) | 0.79 (0.78-0.80) | 0.78 (0.76-0.79) |
| MSE (95% CI) | 0.99 (0.97-1.04) | 1.08 (1.06-1.10) | 1.00 (0.97-1.02) |
| SMAPE (95% CI) | 0.95 (0.94-0.96) | 1.11 (1.10-1.12) | 1.04 (1.02-1.05) |

Supplementary Table 8. Top 10 features with highest SHAP values per model and dataset. Rank agreement through mean Spearman rank correlation and Overlap through Jaccard Index, per dataset and across models and per model and across dataset; For feature descriptions, see Supplementary Table 18. UME = University Medicine Essen; BWH = Brigham and Women’s Hospital; GLM = Generalized linear model; LR = Regularized logistic regression; RF = Random Forest; XGB = XGBoost; CB = CatBoost; TPFN = TabPFN.

| Model | UME Top 10 SHAP Features | BWH Top 10 SHAP Features | Spearman rank correlation across datasets | Jaccard index across datasets |
| --- | --- | --- | --- | --- |
| GLM | fever_change_lag_1,5.04  fever_diff_lag_1,4.80  fourier_max_magnitude,1.62  fever_points_lag_2,1.30  fever_points_lag_1,0.91  predictions_fever_max_we,0.61  time_lag_1_bt_max,0.59  time_lag_1_bt_mean,0.53  fever_variability_lag_1,0.42  trend,0.32 | fourier_max_magnitude,6.19  fever_change_lag_1,4.62  fever_diff_lag_1,4.40  fever_points_lag_2,4.36  fever_points_lag_1,2.89  time_lag_1_bt_max,0.81  predictions_fever_max_we,0.57  time_lag_1_bt_mean,0.51  time_lag_1_krea_max,0.47  fever_variability_lag_1,0.45 | 0.93 | 0.82 |
| LR | predictions_fever_max_we,0.48  time_lag_1_bt_max,0.14  last_bt_fever_lag_1,0.12  time_lag_1_bt_mean,0.08  fever_lag_2_night,0.06  fever_range_lag_2,0.05  fever_percent_lag_1,0.04  time_lag_2_bt_max,0.04  cond_solid,0.03  fever_lag_2_evening,0.01 | predictions_fever_max_we,0.4  time_lag_1_bt_max,0.19  last_bt_fever_lag_1,0.10  time_lag_1_bt_mean,0.07  fever_lag_2_night,0.06  fever_range_lag_2,0.06  cond_solid,0.05  time_lag_2_bt_max,0.05  fever_percent_lag_1,0.05  fever_lag_2_evening,0.01 | 0.95 | 1.0 |
| RF | predictions_fever_max_we,0.12  time_lag_1_bt_mean,0.034  time_lag_1_bt_max,0.02  fever_lag_1_morning,0.02  fever_lag_2_evening,0.01  skewness_lag_all,0.01  fever_variability_lag_2,0.01  time_lag_1_crp_max,0.01  fourier_max_magnitude,0.01  time_lag_2_map_max,0.01 | predictions_fever_max_we,0.10  time_lag_1_bt_mean,0.05  time_lag_1_bt_max,0.02  fever_lag_1_morning,0.02  fever_lag_2_evening,0.01  skewness_lag_all,0.01  time_lag_2_map_max,0.01  fourier_max_magnitude,0.01  time_lag_2_bt_max,0.01  fever_variability_lag_2,0.01 | 0.85 | 0.82 |
| CB | predictions_fever_max_we,0.32  time_lag_1_bt_max,0.13  fever_lag_1_morning,0.09  trend,0.09  time_lag_1_bt_mean,0.09  last_bt_fever_lag_1,0.08  time_lag_2_bt_mean,0.07  fever_lag_2_night,0.06  time_lag_1_heart_rate_max,0.05  fever_percent_lag_1,0.05 | predictions_fever_max_we,0.30  time_lag_1_bt_max, 0.13  fever_lag_1_morning,0.09  time_lag_1_bt_mean,0.08  trend,0.08  fever_points_lag_1,0.08  last_bt_fever_lag_1,0.08  time_lag_2_bt_mean,0.07  time_lag_1_heart_rate_max,0.06  fever_percent_lag_1,0.06 | 0.83 | 0.82 |
| XGB | predictions_fever_max_we,0.29  time_lag_1_bt_max,0.09  time_lag_1_bt_mean,0.06  trend,0.05  fever_lag_1_morning,0.04  time_lag_2_bt_mean,0.03  time_lag_1_map_max,0.02  time_lag_1_heart_rate_max,0.02  time_lag_1_leua_max,0.02  fever_change_lag_1,0.01 | predictions_fever_max_we,0.27  time_lag_1_bt_max,0.10  time_lag_1_bt_mean,0.06  trend,0.05  time_lag_2_bt_mean,0.03  fever_lag_1_morning,0.03  time_lag_1_map_max,0.01  time_lag_1_heart_rate_max,0.01  fever_change_lag_1,0.01  time_lag_1_leua_max,0.01 | 0.98 | 1.0 |
| TPFN | predictions_fever_max_we,0.08  time_lag_1_bt_mean,0.03  time_lag_1_bt_max,0.02  last_bt_fever_lag_1,0.02  fever_percent_lag_1,0.01  trend,0.01  time_lag_2_bt_mean,0.01  fever_lag_1_morning,0.01  time_lag_2_bt_max,0.01  fever_lag_2_night,0.01 | predictions_fever_max_we,0.06  time_lag_1_bt_mean,0.03  time_lag_1_bt_max,0.02  last_bt_fever_lag_1,0.02  fever_percent_lag_1,0.02  fever_points_lag_1,0.01  fourier_max_magnitude,0.01  time_lag_2_bt_mean,0.01  time_lag_1_heart_rate_max,0.01  trend,0.01 | 0.66 | 0.54 |
| Spearman rank correlation across models | 0.40 | 0.42 |  | |
| Jaccard index across models | 0.36 | 0.35 |  |  |

Supplementary Table 9. Top 10 features with highest Permutation Feature Importance values for the XGBoost model and per dataset. For feature descriptions, see Supplementary Table 18. UME = University Medicine Essen; BWH = Brigham and Women’s Hospital; XGB = XGBoost.

| Model | UME Top 10 Permutation Feature Importance, decrease in AUC (95% CI) | | BWH Top 10 Permutation Feature Importance, decrease in AUC (95% CI) | |
| --- | --- | --- | --- | --- |
| XGB | predictions_fever_max_we | 0.084 (0.058-0.097) | predictions_fever_max_we | 0.082 (0.074-0.088) |
|  | time_lag_1_bt_mean | 0.022 (0.014-0.034) | time_lag_1_bt_max | 0.035 (0.026-0.043) |
|  | time_lag_1_bt_max | 0.021 (0.012-0.032) | time_lag_1_bt_mean | 0.028 (0.018-0.039) |
|  | last_bt_fever_lag_1 | 0.006 (0.001-0.011) | last_bt_fever_lag_1 | 0.003 (0.002-0.004) |
|  | fever_lag_1_afternoon | 0.003 (0.001-0.009) | fever_diff_lag_1 | 0.002 (0.002-0.003) |
|  | fever_percent_lag_1 | 0.002 (0.000-0.006) | trend | 0.002 (0.001-0.003) |
|  | fever_change_lag_1 | 0.002 (0.000-0.005) | fever_lag_1_afternoon | 0.001 (0.000-0.002) |
|  | fourier_max_magnitude | 0.001 (0.000-0.004) | fever_change_lag_1 | 0.001 (0.000-0.002) |
|  | fever_lag_2_night | 0.001 (0.000-0.003) | fever_diff_lag_2 | 0.001 (0.000-0.001) |
|  | time_lag_1_leua_max | 0.001 (0.000-0.003) | time_lag_1_heart_rate_max | 0.001 (0.000-0.001) |

Supplementary Table 10. Performance of temperature-focused comparator and feature-ablation models. UME = University Medicine Essen; BWH = Brigham and Women’s Hospital; XGB = XGBoost; LR = Logistic Regression.

| Cohort | UME CV | BWH |
| --- | --- | --- |
|  | Standard temperature summaries only | |
| LR, AUC (95% CI) | 0.70 (0.66-0.73) | 0.72 (CI 0.70-0.73) |
|  | Forecast-derived feature only | |
| XGB, AUC (95% CI) | 0.73 (0.70-0.76) | 0.74 (0.73-0.75) |
|  | Full temperature signal ablation | |
| XGB, AUC (95% CI) | 0.57 (CI 0.54-0.61) | 0.56 (CI 0.55-0.58) |

Supplementary Table 11. Stratified discriminative performance per model during 5-fold nested CV over 20 trials on the UME dataset. UME = University Medicine Essen; GLM = Generalized linear model; LR = Regularized logistic regression; RF = Random Forest; XGB = XGBoost; CB = CatBoost; TPFN = TabPFN.

|  | AUROC (95% CI) | | | | | |
| --- | --- | --- | --- | --- | --- | --- |
|  | GLM | LR | RF | CB | XGB | TPFN |
| Female, n = 379 | 0.73 (0.68-0.78) | 0.74 (0.69-0.79) | 0.74 (0.69-0.79) | 0.78 (0.73-0.83) | 0.79 (0.74-0.84) | 0.82 (0.77-0.86) |
| Male, n = 608 | 0.75 (0.71-0.79) | 0.76 (0.72-0.80) | 0.77 (0.73-0.81) | 0.81 (0.77-0.84) | 0.81 (0.78-0.85) | 0.83 (0.80-0.86) |
| Age < 50, n = 310 | 0.80 (0.75-0.85) | 0.80 (0.75-0.85) | 0.79 (0.73-0.84) | 0.83 (0.78-0.88) | 0.84 (0.79-0.88) | 0.86 (0.82-0.90) |
| Age >= 50 & < 65, n = 380 | 0.72 (0.67-0.77) | 0.73 (0.68-0.78) | 0.76 (0.70-0.81) | 0.79 (0.74-0.84) | 0.79 (0.75-0.84) | 0.82 (0.77-0.86) |
| Age >= 65, n = 297 | 0.69 (0.63-0.75) | 0.70 (0.64-0.76) | 0.73 (0.68-0.79) | 0.77 (0.71-0.82) | 0.78 (0.73-0.83) | 0.79 (0.74-0.84) |
| Hemato. Malign., n = 503 | 0.74 (0.69-0.78) | 0.75 (0.71-0.79) | 0.76 (0.71-0.80) | 0.78 (0.74-0.82) | 0.79 (0.75-0.84) | 0.81 (0.78-0.85) |
| Leukemia, n = 310 | 0.73 (0.68-0.79) | 0.76 (0.70-0.81) | 0.77 (0.71-0.82) | 0.81 (0.77-0.86) | 0.82 (0.77-0.86) | 0.84 (0.79-0.88) |
| Solid tumor, n = 174 | 0.73 (0.65-0.80) | 0.73 (0.66-0.81) | 0.76 (0.69-0.82) | 0.81 (0.75-0.87) | 0.82 (0.76-0.88) | 0.84 (0.78-0.89) |

Supplementary Table 12. Stratified discriminative performance per model during external validation on the BWH dataset. BWH = Brigham and Women’s Hospital; GLM = Generalized linear model; LR = Regularized logistic regression; RF = Random Forest; XGB = XGBoost; CB = CatBoost; TPFN = TabPFN.

|  | AUROC (95% CI) | | | | | |
| --- | --- | --- | --- | --- | --- | --- |
|  | GLM | LR | RF | CB | XGB | TPFN |
| Female, n = 3,156 | 0.71 (0.70-0.73) | 0.75 (0.73-0.77) | 0.75 (0.73-0.77) | 0.77 (0.75-0.79) | 0.77 (0.75-0.78) | 0.78 (0.76-0.79) |
| Male, n = 4,253 | 0.71 (0.70-0.73) | 0.75 ( 0.74-0.77) | 0.75 (0.73-0.76) | 0.77 (0.76-0.79) | 0.77 (0.75-0.78) | 0.78 (0.77-0.79) |
| Age < 50, n = 1,478 | 0.73 (0.71-0.76) | 0.77 (0.75-0.79) | 0.77 (0.74-0.79) | 0.79 (0.76-0.81) | 0.79 (0.76-0.81) | 0.80 (0.78-0.82) |
| Age >= 50 & < 65, n = 2,653 | 0.71 (0.69-0.73) | 0.75 (0.73-0.77) | 0.74 (0.72-0.76) | 0.76 (0.74-0.78) | 0.76 (0.74-0.78) | 0.77 (0.75-0.78) |
| Age >= 65, n = 3,278 | 0.70 (0.68-0.72) | 0.75 (0.73-0.77) | 0.75 (0.73-0.76) | 0.77 (0.75-0.78) | 0.76 (0.74-0.78) | 0.77 (0.76-0.79) |
| Hemato. Malign., n = 1,436 | 0.70 (0.68-0.73) | 0.74 (0.71-0.76) | 0.75 (0.72-0.77) | 0.76 (0.74-0.79) | 0.75 (0.73-0.78) | 0.76 (0.74-0.79) |
| Leukemia, n = 1,764 | 0.73 (0.71-0.75) | 0.78 (0.76-0.80) | 0.78 (0.76-0.80) | 0.79 (0.77-0.81) | 0.79 (0.77-0.81) | 0.80 (0.78-0.82) |
| Solid tumor, n = 4,209 | 0.69 (0.67-0.71) | 0.74 (0.73-0.75) | 0.73 (0.71-0.74) | 0.75 (0.74-0.77) | 0.75 (0.74-0.77) | 0.76 (0.75-0.78) |

Supplementary Table 13. Discriminative performance per model during 5-fold nested CV over 20 trials on the UME dataset and external validation on the BWH dataset under an adapted fever definition threshold of $\geq$38.3 °C. UME = University Medicine Essen; BWH = Brigham and Women’s Hospital; GLM = Generalized linear model; LR = Regularized logistic regression; RF = Random Forest; XGB = XGBoost; CB = CatBoost; TPFN = TabPFN.

|  | University Medicine Essen (UME) | | | | | |
| --- | --- | --- | --- | --- | --- | --- |
|  | GLM | LR | RF | CB | XGB | TPFN |
| AUROC (95% CI) | 0.73 (0.70-0.76) | 0.77 (0.74-0.80) | 0.77 (0.74-0.80) | 0.80 (0.77-0.82) | 0.80 (0.77-0.82) | 0.80 (0.77-  0.83) |
|  | Brigham and Women’s Hospital (BWH) | | | | | |
|  | GLM | LR | RF | CB | XGB | TPFN |
| AUROC (95% CI) | 0.73 (0.72-0.74) | 0.77 (0.76-0.78) | 0.76 (0.75-0.77) | 0.78 (0.77-0.79) | 0.78 (0.76-0.79) | 0.79 (0.78-  0.80) |

Supplementary Table 14. Descriptive overlap between true-negative model predictions and antibiotic escalation or chest CT imaging for the UME cohort. UME = University Medicine Essen.

| Statistic | Antibiotic escalation | Chest CT |
| --- | --- | --- |
| Hospital stays with intervention, n | 365 | 223 |
| Unique patients, n | 328 | 202 |
| True negative predictions, n | 103 | 58 |
| True negative proportion, % | 28.2 | 26.0 |
| Paitent-cluster-bootstrap 95%, CI, % | 23.9-33.0 | 20.7-32.2 |
| Earliest included intervention after antibiotic initiation, h | 38 | 41 |
| Latest included intervention after antibiotic initiation, h | 95 | 92 |

Supplementary Table 15. Data preprocessing steps.

| **Processing step** | **Description** |
| --- | --- |
| Measurement errors | **Body temperature:** Remove data points where $x<23 ^{\circ}C$ or $x>47 ^{\circ}C$ |
|  | **Heart rate:** Remove data points where $x<20 bpm$ or $x>300 bpm$ |
|  | **Mean arterial pressure:** Remove data points where $x<20 mmHg$ or $x>200 mmHg$ |
|  | **Hemoglobin:** Remove data points where $x\leq0 g/dl$ or $x>40 g/dl$ |
|  | **Serum Creatinine:** Remove data points where $x\leq0 mg/dl$ or $x>80 mg/dl$ |
|  | **Pulse Oximetry SpO₂:** Remove data points where $x\leq50 \%$ or $x>100 \%$ |
|  | **Bilirubin:** Remove data points where $x\leq0 mg/dl$ or $x>60 mg/dl$ |
|  | **Leukocytes:** Remove data points where $x\leq0 nl$ or $x>400 nl$ |
|  | **C-reactive Protein:** Remove data points where $x\leq0 mg/dl$ or $x>50 mg/dl$ |
| Duplicates | Remove redundant records where patient identifier, recorded time and recorded value are identical |
| Missing values | Forward-filling of the last observed values within the context window ($\omega_{c}$); remove time series if no values are included in the prediction window ($\omega_{p}$); use of predefined reference values where no previous value was available for forward-filling |
| Scaling | Standard scaling of numerical covariates |
| Categorical values | One-hot encoding of categorical covariates |

Supplementary Table 16. Missing and forward-filled values per engineered feature and cohort. UME = University Medicine Essen. BWH = Brigham and Women’s hospital.

|  | UME cohort, n=987 | | | |
| --- | --- | --- | --- | --- |
| feature | missing_n | missing_pct | available_n | Reference-value imputation required, n |
| time_lag_1_leua_max | 52 | 5.27 | 935 | 12 |
| time_lag_2_leua_max | 48 | 4.86 | 939 | 12 |
| time_lag_2_so2_max | 34 | 3.44 | 953 | 3 |
| time_lag_2_heart_rate_max | 28 | 2.84 | 959 | 5 |
| time_lag_1_so2_max | 27 | 2.74 | 960 | 3 |
| time_lag_1_heart_rate_max | 23 | 2.33 | 964 | 5 |
| time_lag_2_krea_max | 13 | 1.32 | 974 | 6 |
| time_lag_1_krea_max | 10 | 1.01 | 977 | 6 |
| time_lag_2_bili_max | 10 | 1.01 | 977 | 6 |
| time_lag_2_crp_max | 9 | 0.91 | 978 | 2 |
| time_lag_2_hb_max | 7 | 0.71 | 980 | 3 |
| time_lag_1_bili_max | 7 | 0.71 | 980 | 6 |
| time_lag_1_crp_max | 5 | 0.51 | 982 | 2 |
| time_lag_1_hb_max | 4 | 0.41 | 983 | 3 |
| time_lag_2_map_max | 0 | 0.00 | 987 | 0 |
| time_lag_1_bt_mean | 0 | 0.00 | 987 | 0 |
| time_lag_1_bt_max | 0 | 0.00 | 987 | 0 |
| time_lag_1_map_max | 0 | 0.00 | 987 | 0 |
| time_lag_2_bt_max | 0 | 0.00 | 987 | 0 |
| time_lag_2_bt_mean | 0 | 0.00 | 987 | 0 |
|  | BWH cohort, n=7,409 | | | |
| feature | missing_n | missing_pct | available_n | Reference-value imputation required, n |
| time_lag_2_crp_max | 6001 | 81.00 | 1408 | 5162 |
| time_lag_1_crp_max | 5783 | 78.05 | 1626 | 5162 |
| time_lag_2_bili_max | 1143 | 15.43 | 6266 | 728 |
| time_lag_1_bili_max | 963 | 13.00 | 6466 | 728 |
| time_lag_2_krea_max | 49 | 0.66 | 7360 | 18 |
| time_lag_2_leua_max | 35 | 0.47 | 7374 | 6 |
| time_lag_2_hb_max | 35 | 0.47 | 7374 | 6 |
| time_lag_1_krea_max | 29 | 0.39 | 7380 | 18 |
| time_lag_1_leua_max | 16 | 0.22 | 7393 | 6 |
| time_lag_1_hb_max | 16 | 0.22 | 7393 | 6 |
| time_lag_2_heart_rate_max | 0 | 0.00 | 7409 | 0 |
| time_lag_1_so2_max | 0 | 0.00 | 7409 | 0 |
| time_lag_2_map_max | 0 | 0.00 | 7409 | 0 |
| time_lag_1_map_max | 0 | 0.00 | 7409 | 0 |
| time_lag_1_bt_max | 0 | 0.00 | 7409 | 0 |
| time_lag_1_heart_rate_max | 0 | 0.00 | 7409 | 0 |
| time_lag_2_bt_max | 0 | 0.00 | 7409 | 0 |
| time_lag_2_bt_mean | 0 | 0.00 | 7409 | 0 |
| time_lag_1_bt_mean | 0 | 0.00 | 7409 | 0 |
| time_lag_2_so2_max | 0 | 0.00 | 7409 | 0 |

Supplementary Table 17. Reference values used where forward-filling was not available.

| Variable | Value for imputation |
| --- | --- |
| **CRP** mg/dL | **0.3 mg/dL** |
| **Leukocytes** /nL | **7.5 /nL** |
| **Hemoglobin** g/dL | **14.0 g/dL** |
| **S-Creatinine** mg/dL | **0.9 mg/dL** |
| **Total bilirubin** mg/dL | **0.7 mg/dL** |
| **SpO₂** % | **98%** |
| **Heart rate** bpm | **80 bpm** |
| **MAP** mmHg | **85 mmHg** |
| **Body temperature** °C | **37.0°C** |

Supplementary Table 18. Engineered features derived from the initial covariates.

| **Feature** | **Description** | **Datatype** |
| --- | --- | --- |
| cond_hemat | One-hot encoded ICD-10 code for admission diagnosis of other hematological malignancies | boolean |
| cond_leuk | One-hot encoded ICD-10 code for admission diagnosis of leukemia | boolean |
| cond_solid | One-hot encoded ICD-10 code for admission diagnosis of solid tumors | boolean |
| age | Age of patient at admission | integer |
| elixhauser_score | Elixhauser comorbidity score based on ICD-10 codes prior to admission | float |
| sex_male | One-hot encoded if sex of patient is male | boolean |
| length_stay | Length of days from start of hospital stay to first administered antibiotic dosage | float |
| time_lag_1_bt_max | Highest recorded body temperature (°C) 24-48 hours after first administered antibiotic dosage | float |
| time_lag_2_bt_max | Highest recorded body temperature (°C) 0-24 hours after first administered antibiotic dosage | float |
| time_lag_1_bt_mean | Mean recorded body temperature (°C) 24-48 hours after first administered antibiotic dosage | float |
| time_lag_2_bt_mean | Mean recorded body temperature (°C) 0-24 hours after first administered antibiotic dosage | float |
| time_lag_1_krea_max | Highest recorded serum creatinine (mg/dl) 24-48 hours after first administered antibiotic dosage | float |
| time_lag_2_krea_max | Highest recorded serum creatinine (mg/dl) 0-24 hours after first administered antibiotic dosage | float |
| time_lag_1_bili_max | Highest recorded bilirubin (mg/dl) 24-48 hours after first administered antibiotic dosage | float |
| time_lag_2_bili_max | Highest recorded bilirubin (mg/dl) 0-24 hours after first administered antibiotic dosage | float |
| time_lag_1_leua_max | Highest recorded leucocytes (nl) 24-48 hours after first administered antibiotic dosage | float |
| time_lag_2_leua_max | Highest recorded leucocytes (nl) 0-24 hours after first administered antibiotic dosage | float |
| time_lag_1_hb_max | Highest recorded hemoglobin (g/dl) 24-48 hours after first administered antibiotic dosage | float |
| time_lag_2_hb_max | Highest recorded hemoglobin (g/dl) 0-24 hours after first administered antibiotic dosage | float |
| time_lag_1_crp_max | Highest recorded CRP (mg/dl) 24-48 hours after first administered antibiotic dosage | float |
| time_lag_2_crp_max | Highest recorded CRP (mg/dl) 0-24 hours after first administered antibiotic dosage | float |
| time_lag_1_heart_rate_max | Highest recorded heart rate (bpm) 24-48 hours after first administered antibiotic dosage | float |
| time_lag_2_heart_rate_max | Highest recorded heart rate (bpm) 0-24 hours after first administered antibiotic dosage | float |
| time_lag_1_map_max | Highest recorded mean arterial pressure (mmHg) 24-48 hours after first administered antibiotic dosage | float |
| time_lag_2_map_max | Highest recorded mean arterial pressure (mmHg) 0-24 hours after first administered antibiotic dosage | float |
| time_lag_1_so2_max | Highest recorded SO2 (%) 24-48 hours after first administered antibiotic dosage | float |
| time_lag_2_so2_max | Highest recorded SO2 (%) 0-24 hours after first administered antibiotic dosage | float |
| fever_variability_lag_1 | Body temperature variability 24-48 hours after first administered antibiotic dosage | float |
| fever_variability_lag_2 | Body temperature variability 0-24 hours after first administered antibiotic dosage | float |
| last_bt_fever_lag_1 | Last body temperature measurement before prediction window | float |
| fever_diff_lag_1 | Difference in body temperature from first to last measured value 24-48 hours after first administered antibiotic dosage | float |
| fever_diff_lag_2 | Difference in body temperature from first to last measured value 0-24 hours after first administered antibiotic dosage | float |
| fever_percent_lag_1 | Percentage of measured body temperatures at or over fever threshold 24-48 hours after first administered antibiotic dosage | float |
| fever_percent_lag_2 | Percentage of measured body temperatures at or over fever threshold 0-24 hours after first administered antibiotic dosage | float |
| fever_lag_1_evening | Highest recorded body temperature (°C) during evening hours 24-48 hours after first administered antibiotic dosage | float |
| fever_lag_1_afternoon | Highest recorded body temperature (°C) during afternoon hours 24-48 hours after first administered antibiotic dosage | float |
| fever_lag_1_morning | Highest recorded body temperature (°C) during morning hours 24-48 hours after first administered antibiotic dosage | float |
| fever_lag_1_night | Highest recorded body temperature (°C) during night hours 24-48 hours after first administered antibiotic dosage | float |
| fever_lag_2_evening | Highest recorded body temperature (°C) during evening hours 0-24 hours after first administered antibiotic dosage | float |
| fever_lag_2_afternoon | Highest recorded body temperature (°C) during afternoon hours 0-24 hours after first administered antibiotic dosage | float |
| fever_lag_2_morning | Highest recorded body temperature (°C) during morning hours 0-24 hours after first administered antibiotic dosage | float |
| fever_lag_2_night | Highest recorded body temperature (°C) during night hours 0-24 hours after first administered antibiotic dosage | float |
| fever_points_lag_1 | Number of body temperature recordings 24-48 hours after first administered antibiotic dosage | integer |
| fever_points_lag_2 | Number of body temperature recordings 0-24 hours after first administered antibiotic dosage | integer |
| fever_change_lag_1 | Change rate of measured body temperatures 24-48 hours after first administered antibiotic dosage | float |
| fever_change_lag_all | Change rate of measured body temperatures 0-48 hours after first administered antibiotic dosage | float |
| fever_range_lag_1 | Range of measured body temperatures 24-48 hours after first administered antibiotic dosage | float |
| fever_range_lag_2 | Range of measured body temperatures 0-24 hours after first administered antibiotic dosage | float |
| trend | Trend of measured body temperatures 0-48 hours after first administered antibiotic dosage | float |
| skewness_lag_all | Skewness of measured body temperatures 0-48 hours after first administered antibiotic dosage | float |
| fourier_max_magnitude | Maximum fourier transform magnitude of body temperatures 0-48 hours after first administered antibiotic dosage | float |
| predictions_fever_max_we | Chronos-2 based forecasted highest body temperature 48-72 hours after first administered antibiotic dosage | float |

Supplementary Table 19. Settings of Qwen3-Next-80B-A3B-Instruct for feature extraction from clinical notes.

| Item | Text LLM |
| --- | --- |
| Use in study | Clinical-note feature extraction |
| Model | Qwen3-Next-80B-A3B-Instruct |
| Serving backend | vLLM OpenAI-compatible API |
| GPU allocation | 4 NVIDIA GPUs |
| GPU type | H100 |
| Tensor parallelism | 4 |
| Tool-call parser | Hermes |
| Quantization | None |
| Response format | JSON object |
| Temperature | 0.0 |
| Top-p | 1.0 |
| Max tokens | 262,144 |

Supplementary Table 20. Results from repeated-inference stability testing for LLM-based feature extraction across 5 runs.

| **Feature** | **N encounters** | **All-runs exact agreement rate (%)** | **Mean pairwise agreement (%)** |
| --- | --- | --- | --- |
| infection_focus | 100 | 97.0 | 98.0 |
| clinical_trajectory_0_48h | 100 | 98.0 | 98.7 |
| persistent_positive_cultures | 100 | 98.0 | 98.7 |
| documented_resistance | 100 | 98.0 | 98.7 |
| profound_immunosuppression | 100 | 98.0 | 98.7 |
| clinical_impression | 100 | 99.0 | 99.3 |

Supplementary Table 21. Prompt template for LLM-based clinical phenotype extraction.

**System message**

Extract structured labels and return strict JSON only.

**User message**

You are an expert annotator extracting structured labels for one entity.

Domain context:

Clinical phenotype extraction from German hospital notes.

Entity ID: {ENTITY_ID}

Task:

Extract structured phenotypes from the documents to support downstream prediction or analysis.

Rules:

- Use only information explicitly documented in the notes.

- If a fact is not documented or unclear, return unknown; do not guess.

- Keep evidence snippets short, ideally fewer than 20 words each.

- Return only valid JSON. Do not include markdown or explanations.

Field definitions:

- documented_resistance: Microbiology or clinical documentation mentions antimicrobial resistance or reduced susceptibility, such as MRSA, ESBL, VRE, or resistant organism. Allowed values: true|false|unknown.

- clinical_impression: Global clinical status during the relevant period. 1 = fairly okay/stable; 2 = clinically ill, deteriorating, or severe condition. Allowed values: 1|2.

- clinical_trajectory_0_48h: Trend in clinical condition in the first 48h after antibiotic start, if inferable. Allowed values: improving|stable|worsening|unknown.

- persistent_positive_cultures: Repeat cultures remain positive or persistent bacteremia/microbiological positivity is documented. Allowed values: true|false|unknown.

- profound_immunosuppression: Strong immunosuppression is documented, such as intensive chemotherapy, high-dose steroids, transplant, or severe immune suppression. Allowed values: true|false|unknown.

- infection_focus: Main documented or suspected infection source. Allowed values: pneumonia|uti|intraabdominal|line|ssti|cns|endocarditis|unknown|other.

Output schema, using exact keys:

"documented_resistance": {

"value": "true|false|unknown",

"confidence": 0.0,

"evidence": ["..."]

},

"clinical_impression": {

"value": "1|2|unknown ",

"confidence": 0.0,

"evidence": ["..."]

},

"clinical_trajectory_0_48h": {

"value": "improving|stable|worsening|unknown",

"confidence": 0.0,

"evidence": ["..."]

},

"persistent_positive_cultures": {

"value": "true|false|unknown",

"confidence": 0.0,

"evidence": ["..."]

},

"profound_immunosuppression": {

"value": "true|false|unknown",

"confidence": 0.0,

"evidence": ["..."]

},

"infection_focus": {

"value": "pneumonia|uti|intraabdominal|line|ssti|cns|endocarditis|unknown|other",

"confidence": 0.0,

"evidence": ["..."]

}

}

Documents:

{DOCUMENT_TEXT}

Supplementary Table 22. Agreement between LLM-generated features and a clinician reference standard from 30 clinical notes.

| Feature | N | Classes | Largest class prevalence (%) | Agreement (%, 95% CI) | Cohen's κ (95% CI) |
| --- | --- | --- | --- | --- | --- |
| infection_focus | 30 | 6 | 73.33 | 93.33 (78.68-98.15) | 0.86 (0.64-1.00) |
| clinical_trajectory_0_48h | 30 | 4 | 56.67 | 90.00 (74.38-96.54) | 0.82 (0.61-1.00) |
| profound_immunosuppression | 30 | 2 | 86.67 | 90.00 (74.38-96.54) | 0.67 (0.24-1.00) |
| documented_resistance | 30 | 2 | 60.00 | 96.67 (83.33–99.41) | 0.93 (0.77-1.00) |
| clinical_impression | 30 | 2 | 70.00 | 86.67 (70.32–94.69) | 0.64 (0.25-0.91) |

Supplementary Table 23. Implementation settings of the Chronos-2 forecasting model.

| Parameter | Implementation |
| --- | --- |
| Forecasting Framework | AutoGluon-TimeSeries v1.5.0 |
| Forecasting model | Chronos-2, using the Chronos-2 implementation/checkpoint provided with AutoGluon-TimeSeries v1.5.0 |
| Forecasting mode | Zero-shot inference |
| Input variable | Body temperature (°C) |
| Context window | Hospital admission to 48 h after initiation of antibiotic therapy |
| Temporal resolution | 8-h intervals |
| Prediction horizon | 3 × 8-h intervals (48–72 h after antibiotic initiation) |
| Minimum context length | UME: **6** 8-h intervals; BWH: 6 8-h intervals |
| Median context length (IQR) | UME: **[24** (IQR 21-27)**]** 8-h intervals; BWH: **[15 (IQR 12-18)]** 8-h intervals |
| Entirely missing 8-h intervals | Forward-fill and 0-padding for missing values at the beginning of context window |
| Inference unit | **Each hospital stay independently** |
| Inference settings | **Default Chronos-2 inference settings in AutoGluon-TimeSeries v1.5.0** |
| Fine-tuning | None |
| Site-specific adaptation | None |
| External validation | Identical forecasting procedure applied to BWH without model updating or site-specific adaptation |
| Forecast-derived classification feature | Maximum predicted body temperature across the three forecast time points |

Supplementary Table 24. Hyperparameter search spaces for utilized machine learning models.

| Model | Hyperparameter | Range |
| --- | --- | --- |
| Logistic Regression | C | {10-4, ..., 10} (log scale) |
|  | penalty | [l1, l2, elasticnet] (categorical) |
|  | l1_ratio | {0, ..., 1} (uniform) |
| Random Forest | n_estimators | {100, ..., 500} (integer) |
|  | max_depth | {5, ..., 20} (integer) |
|  | min_samples_split | {2, ..., 20} (integer) |
|  | min_samples_leaf | {2, ..., 12} (integer) |
|  | max_features | [sqrt, log2, None] (categorical) |
|  | bootstrap | [True, False] (categorical) |
| XGBoost | subsample | {0.5, ..., 1.0} (uniform) |
|  | colsample_bytree | {0.5, ..., 1.0} (uniform) |
|  | reg_alpha | {1e-6, …, 10} (log scale) |
|  | reg_lambda | {1e-6, …, 10} (log scale) |
|  | max_depth | {3, ..., 8} (integer) |
|  | learning_rate | {0.01, …, 0.3} (log scale) |
|  | n_estimators | {500, ..., 1000} (integer) |
|  | min_child_weight | {1, ..., 10} (integer) |
|  | gamma | {0, ..., 5} (uniform) |
| CatBoost | depth | {3, ..., 7} (integer) |
|  | iterations | {1000, ..., 2000} (integer) |
|  | learning_rate | {0.05, …, 0.2} (log scale) |
|  | l2_leaf_reg | {1e-2, …, 20} (log scale) |
|  | bagging_temperature | {0.0, …, 5.0} (uniform) |
|  | random_strength | {0.0, …, 2.0} (uniform) |
|  | border_count | {32, ..., 255} (integer) |

Supplementary Table 25. Definition of antibiotic escalation strategies used for evaluation.

| After initiation of antibiotic therapy | ATC Code | Classification |
| --- | --- | --- |
| Addition of: | J01F | Macrolides, lincosamides and streptogramins |
|  | J01G | Aminoglycoside antibacterials |
|  | J01M | Quinolone antibacterials |
|  | J01XA | Glycopeptide antibacterials |
|  | J01XX08 | Linezolid |
| Switch to: | J01DH | Carbapenems |

Supplementary File 1. Tripod+AI Checklist.
